# Prometheus, an omics portal for interkingdom comparative genomic analyses

**DOI:** 10.1371/journal.pone.0240191

**Published:** 2020-10-28

**Authors:** Gunhwan Ko, Insu Jang, Namjin Koo, Seong-Jin Park, Sang-Ho Oh, Min-Seo Kim, Jin-Hyuk Choi, Hyeongmin Kim, Young Mi Sim, Iksu Byeon, Pan-Gyu Kim, Kye Young Kim, Jong-Cheol Yoon, Kyung-Lok Mun, Banghyuk Lee, Gukhee Han, Yong-Min Kim

**Affiliations:** 1 Korean Bioinformation Center, Korea Research Institute of Bioscience and Biotechnology, Daejeon, Republic of Korea; 2 Genome Engineering Research Center, Korea Research Institute of Bioscience and Biotechnology, Daejeon, Republic of Korea; Shanghai Jiao Tong University, CHINA

## Abstract

Functional analyses of genes are crucial for unveiling biological responses, genetic engineering, and developing new medicines. However, functional analyses have largely been restricted to model organisms, representing a major hurdle for functional studies and industrial applications. To resolve this, comparative genome analyses can be used to provide clues to gene functions as well as their evolutionary history. To this end, we present Prometheus, a web-based omics portal that contains more than 17,215 sequences from prokaryotic and eukaryotic genomes. This portal supports interkingdom comparative analyses via a domain architecture-based gene identification system and Gene Search, and users can easily and rapidly identify single or entire gene sets in specific pathways. Bioinformatics tools for further analyses are provided in Prometheus or through Bio-Express, a cloud-based bioinformatics analysis platform. Prometheus is a new paradigm for comparative analyses of large amounts of genomic information.

## Introduction

The completion of the Human Genome Project (2003) was not an end but rather a new beginning for further functional genomic analyses. The ENCyclopedia of DNA Elements (ENCODE) was launched to begin investigating the functions of the identified human genes [1]. In addition, large-scale functional studies, such as interactome or network analyses, were performed in model organisms, including *Arabidopsis thaliana*, *Saccharomyces cerevisiae*, and *Drosophila melanogaster*. These efforts accumulated network information on various interactomes and gene functions. These vast amounts of biological information enabled functional studies that contributed to the unveiling of biological responses, cloning of genes of interest, and development of molecular markers for model organisms or medicines in humans [2, 3]. Thus, the trend of functional analyses has been transferred from candidate gene research to genome-wide research. However, this flood of information has largely been restricted to model organisms, and it has been challenging for researchers to apply these data to newly sequenced genomes.

Since next-generation sequencing (NGS) technology was developed in the mid-2000s, an enormous amount of genomic information has been analyzed and amassed in public databases. As the numbers of sequenced genomes increased, many tools and pipelines were developed to investigate gene functions, identify gene families, and perform comparative genomic analyses. However, the application of comparative analyses is restricted to functional gene annotations and newly sequenced genome analyses. Newly sequenced genomes are initially compared to those that have previously been analyzed, including genomes of closely related species, to provide information on genome structure changes and gene repertoires. Such comparisons can also predict gene paralogs, which are genes related by duplication events, or orthologs, which are those related by speciation events [4–6]. As orthologs tend to be more similar in function than paralogs [7], they are widely used for functional gene annotations [8]. Moreover, recent gene-of-interest studies that include multigenome orthologs offer insight into their mechanisms for adapting to the environment [9, 10]. However, these comparative genomic analyses were performed at genome-, genus-, or kingdom-wide [11–13] levels, thereby restricting comparisons at the species, family, or order level. To understand the evolution of genes of interest more precisely, interkingdom analyses are needed, particularly because many genes in eukaryotic genomes have universal common ancestors in *Bacteria* and *Archaea* [14].

Here, we report Prometheus (https://prometheus.kobic.re.kr), an omics portal for interkingdom comparative genomic analyses. We collected 17,215 genome assemblies from 16,730 species and constructed four primary databases to provide basic genome information, with more detailed information on individual genes provided in secondary databases. Researchers can then access detailed information on genes of interest, such as gene structure, domain architecture, subcellular localization, orthologs, and paralogs, as well as their sequences. In particular, Prometheus provides Gene Search to identify genes of interest based on their domain architectures from prokaryotes to eukaryotes and to perform various comparative analyses, such as comparison of chromosome sequences, sequence alignment, and phylogenetic analyses. Furthermore, researchers can perform various bioinformatics analyses with these and their own sequencing data in a cloud-based platform, Bio-Express. Prometheus presents a new paradigm for genome research, from single genes of interest to entire gene pathways.

## Methods

### Web interface

Prometheus provides data searches, configuration of data analyses, data visualization, and storage of user data. The interface is implemented using Hypertext Markup Language (HTML) and cascading style sheets (CSS) and uses a jQuery JavaScript library (jQuery) to modify web page contents. To visualize data, dynamic web interface is constructed by Asynchronous JavaScript and XML (Ajax) using JavaScript Object Notation (JSON) data format. Furthermore, the genome browser was constructed using Scalable Vector Graphics (SVG), and the phylogenetic viewer was constructed using JavaScript. The web interface of Prometheus supports cross-browsing.

#### Construction of taxonomy combined heatmap of photolyase/cryptochrome family

Sequences for the photolyase/cryptochrome family of genes from different species in previous study [15] were collected and domain architectures were investigated using InterProScan v5.0. Each of the subtypes reported in previous studies were investigated using Gene Search in Prometheus. The numbers of each of the subfamily genes were calculated for individual species and visualized as a heatmap using R scripts. The taxonomic tree was constructed using phyloT in iTOL [16], an online tool that generates phylogenetic trees based on the NCBI taxonomy. Finally, the taxonomic tree and heatmap were combined using Adobe Illustrator.

### Bioinformatics analysis using a cloud-based analysis system, Bio-Express

LAST [17], BLAST [18], Clustal Omega [19], MUSCLE [20] and InterPro [21] programs are run in the hybrid-cluster system, Bio-Express. To support further genomic analyses using personal data such as RNA-seq, ChIP-seq, or genome resequencing data, Prometheus links to Bio-Express, and users can perform further various genomic analyses using personal data in My Gene and various analysis pipelines in Bio-Express. Bio-Express is constructed by Hadoop to support high-speed analysis of a large amount of data. To maintain a large data sets, Prometheus uses HDFS to store the data divided by optimized block sizes into various computer servers. This storage system can maintain three copies of user data and provides stable data storage to reduce risk of data loss. The web server of Prometheus transmits tasks, progress and results of data analysis to the Bio-Express server using Apache thrift library-based Remote Procedure Call (RPC) and receives results in JSON format. The results of genomic analyses are stored in HDFS and downloaded in the web browser using HTTP. In the case of large amounts of data, users can download their data using GBox (High-Speed Data Transmission), a high-speed file transmission software using TCP/IP, and transferred user data are stored in HDFS.

### Database construction

The database of primary and secondary data tables in Prometheus was constructed using the MySQL database management system. In the database, primary data tables were created through data in opened in five public databases, and secondary data tables were constructed by parsing results of bioinformatics tools such as InterProScan, OrthoMCL [22], MultiLoc2 [23] and TargetP [24]. Detailed methods for database construction are described in Supplemental Note Section 1.

## Results

### Concept and construction of Prometheus

**Fig 1:**
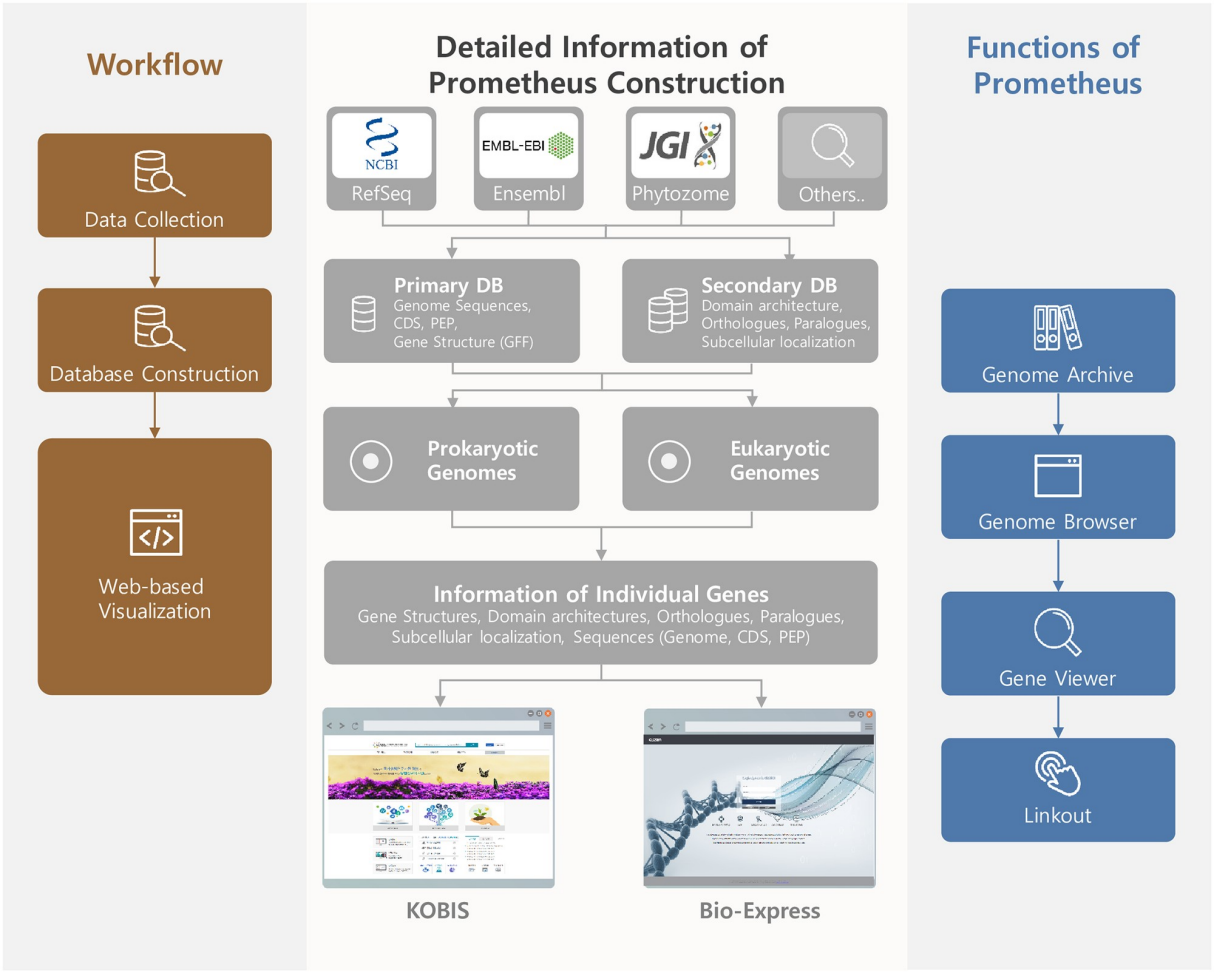
Concept and construction progress of Prometheus. Schematic showing the workflow for constructing Prometheus (left), detailed information for each stage (middle), and the functions available within Prometheus (right).

Prometheus (http://prometheus.kobic.re.kr) provides an integrated pipeline for interkingdom comparative genomic analyses and comprises four major sections, Genome Archive, Gene Search, Bio-Express, and Genome Analysis. Users can identify genes of interest using Gene Search and investigate their domain architectures using InterPro in Genome Analysis. Furthermore, users can obtain additional species information via accessing the Korean Bioresource Information System (KOBIS) or perform further analyses by accessing the cloud-based Bio-Express (Fig 1).

**Fig 2:**
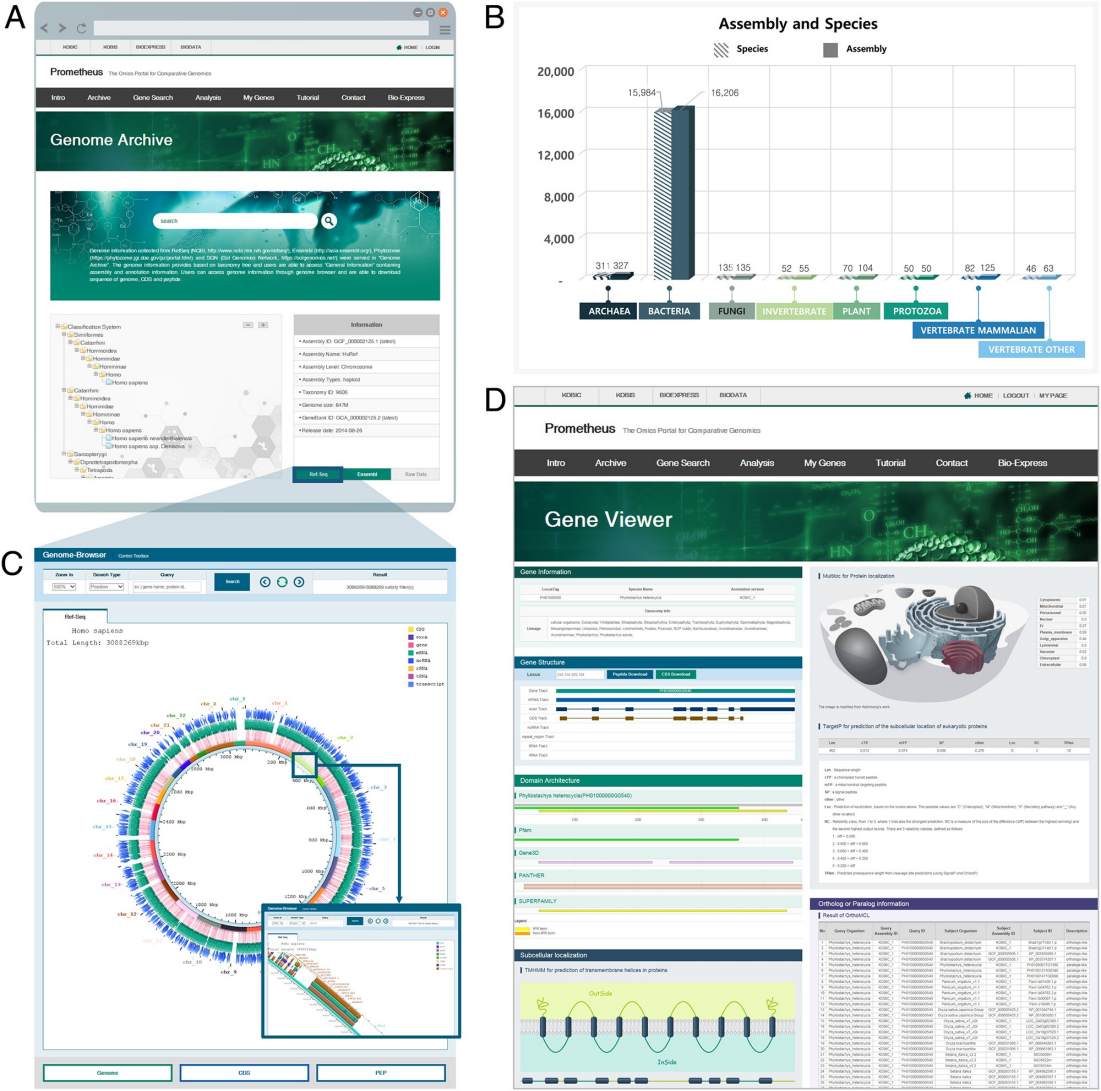
Construction of primary and secondary databases. (A) Screenshot of the Genome Archive page. (B) The numbers of species and genome versions used for the construction of Prometheus. (C) Screenshot of the Genome Browser of Prometheus. A region of the human genome (HGP 38) is shown in the inset. (D) Screenshot of the Gene Viewer, which provides detailed information of individual genes. Gene structure, domain architecture, subcellular localization, and orthologous and paralogous genes are shown in each panel.

To establish Prometheus, 17,215 genome assemblies from 16,730 species were collected and stored in four primary databases. The genomic information in Genome Archive (Fig 2A and Table 1) is arranged by taxonomic rank (obtained from NCBI), which users can access by clicking the species or common name in the taxonomic tree or using a key word search. This general information provides details on genome assembly, annotation, and taxonomy. In eukaryotic genomes, distinct versions of genome assembly and annotation were provided, and so each version is stored separately (Fig 2B and S1 Table in S1 File). Prokaryotic genomic information is separated by strain to support metagenomics analyses. Genomes were classified according to criteria from RefSeq, which provided most of the genomic data (S1 Table in S1 File), to construct the database and to visualize the genomic information. In total, 435 eukaryotic genomes, 15,984 prokaryotic genomes, and 311 archaea genomes were collected and assembled into the four primary databases containing information on assembled genomes, general feature formats (GFFs), coding sequences (CDSs), and protein sequences, for a total 213,478,449 records (S2 Table in S1 File). Five secondary databases containing information of subcellular localization, domains, and homologs in the same or different species were constructed (S3–S5 Tables in S1 File). Taxonomic information in Genome Archive is stored in a taxonomy database, and general information of genome assembly and annotation is stored in a genome report database. In total, 11 databases were constructed with 1,163,053,603 records (S3–S5 Tables in S1 File).

**Table 1:** Statistics of species and their genomic sequences in Prometheus.

Kingdom	Numbers of species	Numbers of genomic sequences
Archaea	311	327
Bacteria	15,984	16,358
Fungi	135	135
Animalia	180	241
Plantae	70	104
Protista	50	50
**Total**	**16,730**	**17,215**

We investigated other genome databases, such as CoGe [25], Ensembl [26], PLAZA 4.0 [27], and MicrobesOnline [28], to compare genome contents and data types (S6 Table in S1 File). CoGe and Ensembl provide more genome assemblies than Prometheus. However, CoGe provides only total numbers of genomes. Ensembl provides ortholog/paralog information between two genomes while Prometheus provides ortholog/paralog information among multiple genomes in same family. PLAZA 4.0 and MicrobesOnline are focused on comparative genomics analysis in plants or bacteria and archaea and do not provide subcellular localization information. The most remarkable features of Prometheus are its domain architecture-based gene search and availability of individual gene domain architecture information in Genome Browser or Gene Search. Thus, users can carry out interkingdom comparative analysis using large sequence data sets.

General information on individual genomes is obtained using Genome Browser (Fig 2C), with zoom in/out functions ranging from 1× to 10× and a gene search function by position or gene name. Users can access and download the individual gene’s information (CDS, and peptide sequence) by key word search or by clicking within the Genome Browser. Detailed information on individual genes is provided in Gene Viewer (Fig 2D), and users can access the Genome Browser or result pages in Gene Search. Bioinformatics analyses, including InterPro [21], OrthoMCL [22], MultiLoc2 [23], and TargetP [24], were performed using protein sequences of each species to construct six secondary databases, which are presented in separate sections within the Gene Viewer (Fig 2D). Using Gene Viewer, researchers can save time by accessing various gene information more easily instead of visiting individual websites to determine subcellular localization, putative orthologs or paralogs and domain architectures.

### Analyses of transcriptional factors and TCA cycle in gene search

**Fig 3:**
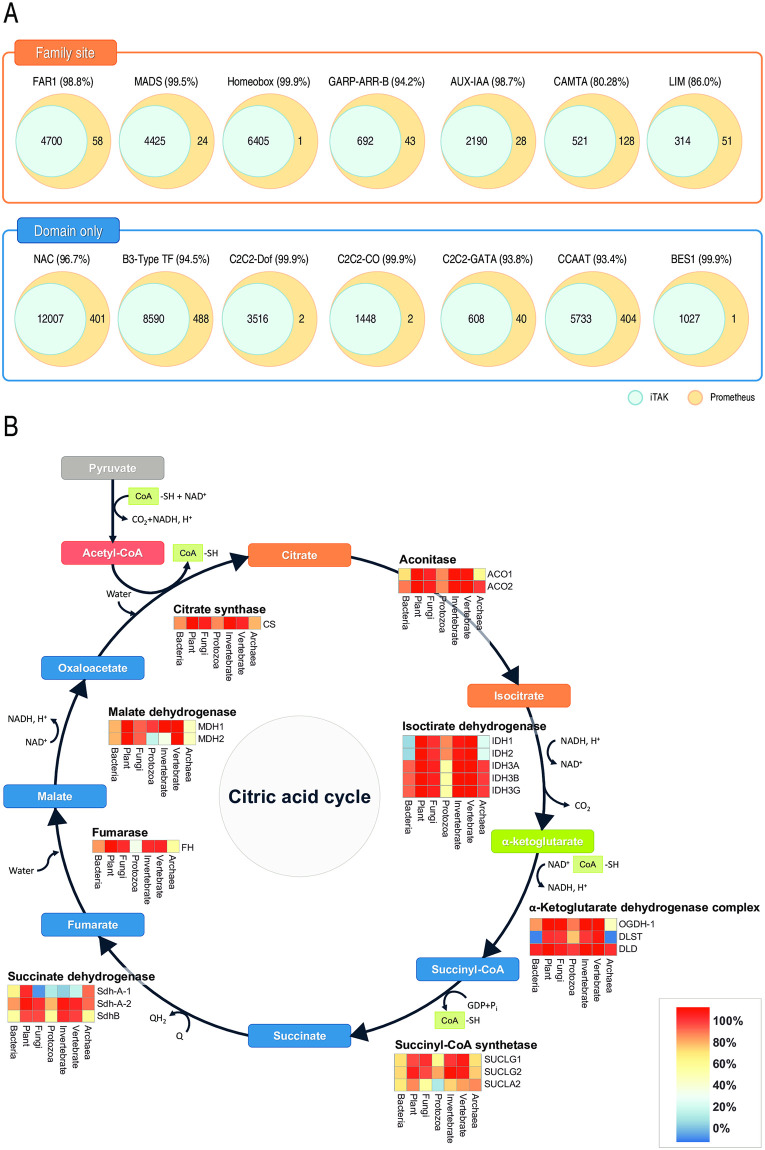
Identification of TFs and genes in the TCA using gene search. (A) Validation of identified TFs using the iTAK pipeline. (B) Human TCA cycle genes were investigated and used for further analysis. The ratios of each gene are shown as heatmaps.

The major function of Prometheus is to perform interkingdom comparative analyses. To support this objective, secondary databases containing information on domain architectures and orthologs/paralogs of individual genes were constructed. Prometheus has totally contained over 60 million unique proteins which was extracted from primary database such as Ensembl, Phytozome, Refseq, Solgenomics and the others (S7 Table in S1 File). Domain architectures of individual proteins were analyzed using InterPro and were shown as IPR terms. Thus, users can identify genes of interest using Gene Search by typing their domain architectures using IPR terms with high performance (S8 Table in S1 File). We validated the utility of Prometheus by performing an interkingdom investigation of transcription factors (TFs) and genes involved in the TCA cycle using Gene Search (Fig 3 and S7 and S8 Tables in S1 File). A pipeline (iTAK v1.7) [29] was used to identify plant TFs and classify protein kinases. TFs, transcriptional regulators (TRs), and kinases were identified by consensus rules mainly summarized from PlnTFDB [30], PlantTFDB [31] with families from PlantTFact [32], and AtFDB [29]. Domain architectures of each TF were investigated using InterProScan, and their domain architectures depicted by IPR terms were used for further analyses using Gene Search. To provide additional information about identified genes, the number of domain subtypes are depicted in a summary table in Gene Search and as a header of sequence data in a FASTA file (S1 Fig in S1 File). Users can categorize identified genes into each subtype. We identified and validated 79,960 genes from 15 gene families using the iTAK pipeline v1.7 [29] (Fig 3A and S9 Table in S1 File). The accuracy of our Gene Search ranged from 86.03% to 99.98%, with an average accuracy of 96.41%. High rates of accuracy were observed for genes encoding TFs containing significant IPR terms, such as FAR1, MADS, NAC, or Dof domains, whereas those for TFs without significant IPR terms, such as B3-type TFs or CAMTA, showed lower rates. Thus, these data suggest that specific IPR terms or exact domain architectures are required to enhance the accuracy of Gene Search.

Genes involved in the TCA cycle were further investigated with Gene Search to demonstrate the potential for applying comparative genomics at the pathway level. As the TCA cycle is a fundamental metabolic pathway for survival in prokaryotes and eukaryotes, we selected this for an interkingdom comparative genomic analysis. A total of 435,044 genes were identified from 20 individual genes in the TCA cycle using Gene Search, and the ratios of species harboring each gene in the TCA cycle were shown as heatmaps (Fig 3B and S10 Table in S1 File). These results showed that some genes, such as those encoding isocitrate dehydrogenase (IDH1 and IDH2) and malate dehydrogenase (MDH1 and MDH2) evolved in a lineage-specific manner. Furthermore, the results show the lineage-specific rates of functionally redundant genes, such as those encoding succinate dehydrogenase and succinyl-CoA synthase. This investigation of the TCA cycle also provided information on the gene repertoires and the evolution of the TCA cycle in each kingdom. Thus, Prometheus provides information for evolutionary studies of single genes or those in specific pathways, including the distributions and rates of genes, as well as repertoires of gene orthologs in pathways. In addition, Prometheus provides the domain architectures of genes as well as their CDSs and/or peptide sequences.

### Tools for comparative analyses and personalized management system via My Genes in Prometheus

To support comparative analyses in Prometheus, essential tools such as LAST [17] (a program for comparing sequences at the chromosome level), BLAST [18], and InterPro [21] are provided in Genome Analysis (S2 Fig in S1 File). Users can monitor the progress of analysis in a personalized page, My Genes (S3 Fig in S1 File), and download the result files from each program via a file menu. In the case of data from InterProScan, the result file is shown in a graphic format and results are downloaded in a .tsv file format (S4 Fig in S1 File). Thus, users can investigate domain architectures of genes of interest and perform interkingdom identification using Gene Search.

**Fig 4:**
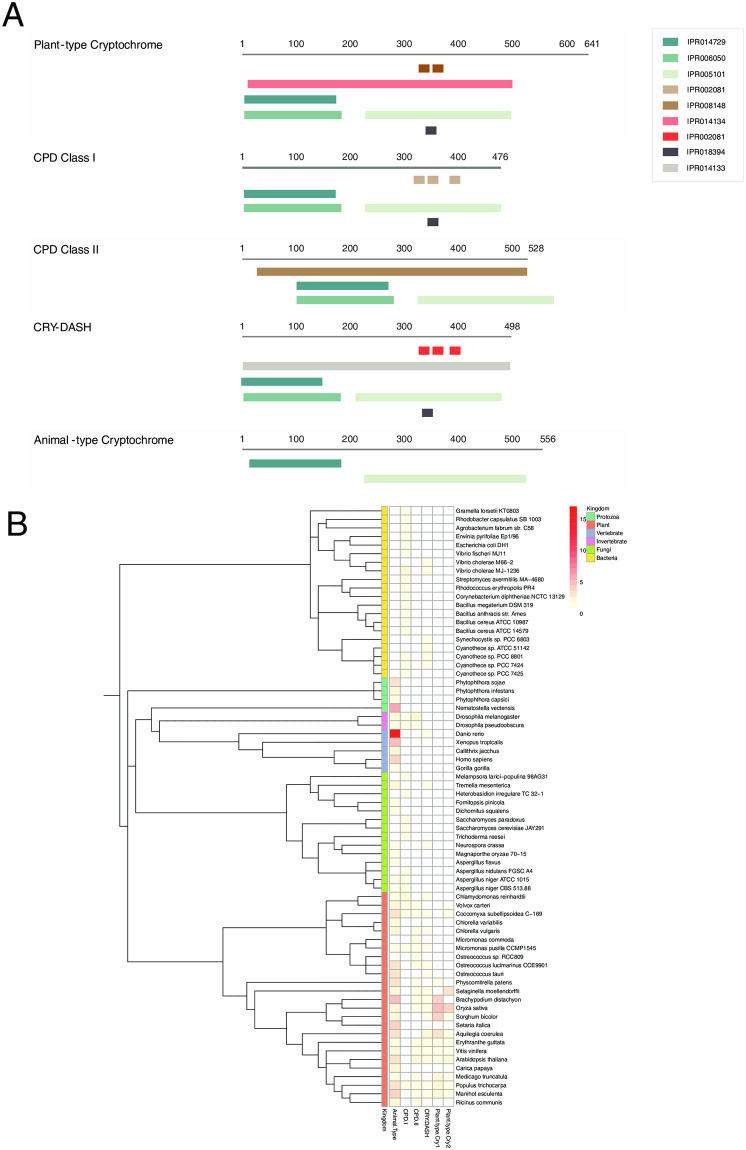
Interkingdom comparative analysis of the photolyase/cryptochrome gene family. (A) Domain architectures of the photolyase/cryptochrome gene family. (B) Taxonomic distribution of photolyase/cryptochrome genes.

We performed a comparative analysis of genes in the photolyase/cryptochrome family using a gene set from a previous study [15] as a control (Fig 4 and S11 Table in S1 File). The domain architectures of photolyase/cryptochrome subfamilies are the same and family IPR terms are different (Fig 4A), enabling a more accurate identification of each subfamily. The results also indicated lineage-specific distributions of photolyase/cryptochrome gene families in each kingdom. Furthermore, the gene repertoires of each subgroup of these families are shown in a combined taxonomy heatmap (Fig 4B), demonstrating lineage-specific evolution and the expansion of subgroups at the species level. These data demonstrate that Gene Search and bioinformatics tools in Genome Analysis in Prometheus support interkingdom comparative analyses. In summary, Prometheus provides the bioinformatics tools essential for comparative analyses, and users can combine these tools with interkingdom comparative analyses in Gene Search to unveil gene function or the evolution of genes/gene families.

### Further genomic analyses using Bio-Express with personalized data via My Genes

Personal data, such as RNA-seq or ChIP-seq data, and sequence data downloaded from Prometheus (e.g., genome, CDS, and peptide) in Genome Archive or FASTA files from Gene Search can be uploaded and stored in My Genes (S3 Fig in S1 File) and further analyzed using the cloud-based Bio-Express platform (https://www.bioexpress.re.kr/). Bio-Express system consists of cluster nodes for bioinformatics analysis, Hadoop Distributed File System (HDFS) storage for data deposition, cache solutions, and a distributed task scheduler (S5 Fig in S1 File). The Bio-Express hardware system consists of 900 core CPUs, 9.1 TB of memory, and 1.5 PB of disk storage in total [33]. Programs for bioinformatics analysis in Bio-Express are modularized and shown as icons (S6 Fig in S1 File). Users can construct their own analysis pipelines by selecting and linking each modularized program using arrows and programs were provided in Bio-Express were summarized in S12 Table in S1 File. We performed a transcriptomic analysis in Bio-Express using the genome of *Hibiscus syriacus* [6] and RNA-seq data. For this, TopHat2 [34] and Cufflinks [35] programs were used, and genes differentially expressed in tissues from a previous study were identified and visualized as a heatmap (S7 Fig in S1 File). Thus, users can perform bioinformatics analyses with personal data in My Genes by linking to Bio-Express. This combination of Prometheus and Bio-Express can provide convenient and user-friendly analysis conditions for non-bioinformatician scientists.

## Discussion

Since NGS technology was developed and applied to biology, vast amounts of genomic data have accumulated. With these data, comparative analyses of species or genes can be performed to unveil gene function or evolution. For instance, the evolution of pungency in peppers was discovered by a comparative analysis with tomato and potato genomes [5]. However, only a small number of biologists can perform these comparative analyses using bioinformatics tools. Indeed, the accessibility of bioinformatics analysis is currently a major hurdle for ongoing biologic research. Thus, we constructed Prometheus, a web-based omics portal for interkingdom comparative genomic analyses. Biologists can identify genes or gene families of interest using the domain architectures in Gene Search. Genes from multigene families containing various domain architectures can be detected, such as for the photolyase/cryptochrome family [15] and the nucleotide-binding leucine-rich repeat gene family [36]. Additional subtype information of identified genes is provided in the headers for their sequences in FASTA files.

The goal of combining kingdom-wide gene identification with subtype information is to provide evolutionary insight by detecting lineage-specific subtypes or subtype distribution patterns, as exemplified by the analysis of gene subtypes involved in the TCA cycle. Moreover, users can perform comparative analyses of single genes as well as sets of genes involved in specific signaling pathways. We found that genes containing specific domains showed high rates of accuracy in domain architecture-based Gene Search in Prometheus. However, the accuracy was reduced for genes without specific IPR terms, which is a limitation of domain architecture-based gene search systems using InterPro or the pfam database. Nevertheless, this limitation will be minimized as Prometheus is updated with new releases of these databases.

To support comparative analyses, Prometheus incorporates various tools, such as LAST, Clustal Omega, and Phylogeny viewer, in Genome Analysis. This is a valuable addition, as there are currently few web sites for comparative analyses with large restrictive or functionally important gene families, such as TFs. For TFs in plants, there are two representative web sites, PlnTFDB [30] and PlantTFDB [31], but their gene repertoires differ due to their rules for indemnification of TFs [37]. Prometheus clears this particular hurdle via its domain architecture-based Gene Search system, thereby providing biologists with a powerful comparative analysis platform with various tools for further studies.

Prometheus provides information to users on individual genomes assigned by taxonomy in Genome Archive via Genome Browser. Here, users can download the genomic and peptide sequences and CDSs as well as upload their own data for further analyses in Prometheus or the cloud-based Bio-Express platform. Furthermore, users can access detailed information on genes of interest in the Gene Viewer page. The connection with Bio-Express enables Prometheus to provide various bioinformatics tools and allows biologists to analyze their own data in same platform. Thus, unlike other comparative genomics portals or platforms, Prometheus provides tools not only for comparative analyses but also for genomic analyses, such as transcriptome or resequencing analyses.

## Conclusion

Prometheus is an integrated platform for interkingdom comparative genomic analyses with additional support for other genomic analyses with the user’s own data. Users can identify genes of interest based on their domain architecture using Gene Search as well as conventional methods using sequence similarity from domains Archaea, Bacteria, and Eukarya. The domain architecture-based gene search can provide precise gene sets compared to sequence similarity gene sets. Users can investigate detailed information including domain architectures, subcellular localization, and putative orthologs or paralogs of individual genes identified by Gene Search in Gene Viewer and predict their putative functions. Users can also carry out interkingdom analyses of large data sets for evolutionary studies. Analysis tools such as LAST, Clustal Omega, and Phylogeny viewer will support such studies. Thus, Prometheus offers biologists a new paradigm for comparative genome analyses and evolution studies. The platform and InterPro version will be updated annually with newly sequenced genomes to ensure that broad and precise data are available to researchers. Furthermore, newly developed tools for comparative genomic analyses will continue to be added to support various analyses. Finally, visualization of domain subtype architectures identified by Gene Search is now being developed and will be available for updates in the near future.

## Supporting information

S1 File (DOCX)

## References

[1] Consortium EP. An integrated encyclopedia of DNA elements in the human genome. Nature. 2012;489(7414):57–74. Epub 2012/09/08. 10.1038/nature11247. . 22955616 PMC3439153

[2] Arabidopsis Interactome Mapping C. Evidence for network evolution in an Arabidopsis interactome map. Science. 2011;333(6042):601–7. 10.1126/science.1203877. . 21798944 PMC3170756

[3] Rolland T, Tasan M, Charloteaux B, Pevzner SJ, Zhong Q, Sahni N, et al A proteome-scale map of the human interactome network. Cell. 2014;159(5):1212–26. 10.1016/j.cell.2014.10.050. . 25416956 PMC4266588

[4] Tomato Genome C. The tomato genome sequence provides insights into fleshy fruit evolution. Nature. 2012;485(7400):635–41. 10.1038/nature11119. . 22660326 PMC3378239

[5] Kim S, Park M, Yeom SI, Kim YM, Lee JM, Lee HA, et al Genome sequence of the hot pepper provides insights into the evolution of pungency in Capsicum species. Nat Genet. 2014;46(3):270–8. 10.1038/ng.2877. . 24441736

[6] Kim YM, Kim S, Koo N, Shin AY, Yeom SI, Seo E, et al Genome analysis of Hibiscus syriacus provides insights of polyploidization and indeterminate flowering in woody plants. DNA Res. 2017;24(1):71–80. 10.1093/dnares/dsw049. . 28011721 PMC5381346

[7] Altenhoff AM, Studer RA, Robinson-Rechavi M, Dessimoz C. Resolving the ortholog conjecture: orthologs tend to be weakly, but significantly, more similar in function than paralogs. PLoS Comput Biol. 2012;8(5):e1002514 10.1371/journal.pcbi.1002514. . 22615551 PMC3355068

[8] Mi H, Muruganujan A, Casagrande JT, Thomas PD. Large-scale gene function analysis with the PANTHER classification system. Nat Protoc. 2013;8(8):1551–66. 10.1038/nprot.2013.092. . 23868073 PMC6519453

[9] Peng T, Lin J, Xu YZ, Zhang Y. Comparative genomics reveals new evolutionary and ecological patterns of selenium utilization in bacteria. The ISME journal. 2016 10.1038/ismej.2015.246. . 26800233 PMC5029168

[10] Wang X, Guo H, Wang J, Lei T, Liu T, Wang Z, et al Comparative genomic de-convolution of the cotton genome revealed a decaploid ancestor and widespread chromosomal fractionation. The New phytologist. 2016;209(3):1252–63. 10.1111/nph.13689. . 26756535

[11] Goodstein DM, Shu S, Howson R, Neupane R, Hayes RD, Fazo J, et al Phytozome: a comparative platform for green plant genomics. Nucleic Acids Res. 2012;40(Database issue):D1178–86. 10.1093/nar/gkr944. . 22110026 PMC3245001

[12] Herrero J, Muffato M, Beal K, Fitzgerald S, Gordon L, Pignatelli M, et al Ensembl comparative genomics resources. Database: the journal of biological databases and curation. 2016;2016 10.1093/database/bav096. . 26896847 PMC4761110

[13] Haeussler M, Zweig AS, Tyner C, Speir ML, Rosenbloom KR, Raney BJ, et al The UCSC Genome Browser database: 2019 update. Nucleic Acids Res. 2019;47(D1):D853–D8. Epub 2018/11/09. 10.1093/nar/gky1095. . 30407534 PMC6323953

[14] Theobald DL. A formal test of the theory of universal common ancestry. Nature. 2010;465(7295):219–22. 10.1038/nature09014. . 20463738

[15] Kim YM, Choi J, Lee HY, Lee GW, Lee YH, Choi D. dbCRY: a Web-based comparative and evolutionary genomics platform for blue-light receptors. Database: the journal of biological databases and curation. 2014;2014(0):bau037. 10.1093/database/bau037. . 24816342 PMC4016680

[16] Letunic I, Bork P. Interactive tree of life (iTOL) v3: an online tool for the display and annotation of phylogenetic and other trees. Nucleic Acids Res. 2016;44(W1):W242–5. 10.1093/nar/gkw290. . 27095192 PMC4987883

[17] Kielbasa SM, Wan R, Sato K, Horton P, Frith MC. Adaptive seeds tame genomic sequence comparison. Genome research. 2011;21(3):487–93. 10.1101/gr.113985.110. . 21209072 PMC3044862

[18] Altschul SF, Gish W, Miller W, Myers EW, Lipman DJ. Basic local alignment search tool. J Mol Biol. 1990;215(3):403–10. 10.1016/S0022-2836(05)80360-2. . 2231712

[19] Sievers F, Higgins DG. Clustal Omega, accurate alignment of very large numbers of sequences. Methods in molecular biology. 2014;1079:105–16. Epub 2013/10/31. 10.1007/978-1-62703-646-7_6. . 24170397

[20] Edgar RC. MUSCLE: multiple sequence alignment with high accuracy and high throughput. Nucleic Acids Res. 2004;32(5):1792–7. 10.1093/nar/gkh340. . 15034147 PMC390337

[21] Mitchell AL, Attwood TK, Babbitt PC, Blum M, Bork P, Bridge A, et al InterPro in 2019: improving coverage, classification and access to protein sequence annotations. Nucleic Acids Res. 2019;47(D1):D351–D60. Epub 2018/11/07. 10.1093/nar/gky1100. . 30398656 PMC6323941

[22] Li L, Stoeckert CJ Jr., Roos DS. OrthoMCL: identification of ortholog groups for eukaryotic genomes. Genome research. 2003;13(9):2178–89. 10.1101/gr.1224503. . 12952885 PMC403725

[23] Blum T, Briesemeister S, Kohlbacher O. MultiLoc2: integrating phylogeny and Gene Ontology terms improves subcellular protein localization prediction. BMC Bioinformatics. 2009;10:274 10.1186/1471-2105-10-274. . 19723330 PMC2745392

[24] Emanuelsson O, Brunak S, von Heijne G, Nielsen H. Locating proteins in the cell using TargetP, SignalP and related tools. Nat Protoc. 2007;2(4):953–71. 10.1038/nprot.2007.131. . 17446895

[25] Lyons E, Freeling M. How to usefully compare homologous plant genes and chromosomes as DNA sequences. The Plant Journal. 2008;53(4):661–73. 10.1111/j.1365-313X.2007.03326.x. 18269575

[26] Herrero J, Muffato M, Beal K, Fitzgerald S, Gordon L, Pignatelli M, et al Ensembl comparative genomics resources. Database. 2016;2016 10.1093/database/bav096. PMC4761110 26896847

[27] Van Bel M, Diels T, Vancaester E, Kreft L, Botzki A, Van de Peer Y, et al PLAZA 4.0: an integrative resource for functional, evolutionary and comparative plant genomics. Nucleic Acids Research. 2017;46(D1):D1190–D6. 10.1093/nar/gkx1002. PMC5753339 29069403

[28] Dehal PS, Joachimiak MP, Price MN, Bates JT, Baumohl JK, Chivian D, et al MicrobesOnline: an integrated portal for comparative and functional genomics. Nucleic Acids Res. 2010;38(Database issue):D396–400. Epub 2009/11/13. 10.1093/nar/gkp919. . 19906701 PMC2808868

[29] Yilmaz A, Mejia-Guerra MK, Kurz K, Liang X, Welch L, Grotewold E. AGRIS: the Arabidopsis Gene Regulatory Information Server, an update. Nucleic Acids Res. 2011;39(Database issue):D1118–22. 10.1093/nar/gkq1120. . 21059685 PMC3013708

[30] Perez-Rodriguez P, Riano-Pachon DM, Correa LG, Rensing SA, Kersten B, Mueller-Roeber B. PlnTFDB: updated content and new features of the plant transcription factor database. Nucleic Acids Res. 2010;38(Database issue):D822–7. 10.1093/nar/gkp805. . 19858103 PMC2808933

[31] Jin J, Zhang H, Kong L, Gao G, Luo J. PlantTFDB 3.0: a portal for the functional and evolutionary study of plant transcription factors. Nucleic Acids Res. 2014;42(Database issue):D1182–7. 10.1093/nar/gkt1016. . 24174544 PMC3965000

[32] Dai X, Sinharoy S, Udvardi M, Zhao PX. PlantTFcat: an online plant transcription factor and transcriptional regulator categorization and analysis tool. BMC Bioinformatics. 2013;14:321 10.1186/1471-2105-14-321. . 24219505 PMC4225725

[33] Ko G, Kim PG, Yoon J, Han G, Park SJ, Song W, et al Closha: bioinformatics workflow system for the analysis of massive sequencing data. BMC Bioinformatics. 2018;19(Suppl 1):43 Epub 2018/03/06. 10.1186/s12859-018-2019-3. . 29504905 PMC5836837

[34] Kim D, Pertea G, Trapnell C, Pimentel H, Kelley R, Salzberg SL. TopHat2: accurate alignment of transcriptomes in the presence of insertions, deletions and gene fusions. Genome Biol. 2013;14(4):R36 Epub 2013/04/27. 10.1186/gb-2013-14-4-r36. . 23618408 PMC4053844

[35] Trapnell C, Hendrickson DG, Sauvageau M, Goff L, Rinn JL, Pachter L. Differential analysis of gene regulation at transcript resolution with RNA-seq. Nature Biotechnology. 2012;31:46 10.1038/nbt.2450 https://www.nature.com/articles/nbt.2450#supplementary-information. 23222703 PMC3869392

[36] Seo E, Kim S, Yeom S-I, Choi D. Genome-Wide Comparative Analyses Reveal the Dynamic Evolution of Nucleotide-Binding Leucine-Rich Repeat Gene Family among Solanaceae Plants. Frontiers in plant science. 2016;7(1205). 10.3389/fpls.2016.01205. PMC4978739 27559340

[37] Zheng Y, Jiao C, Sun H, Rosli HG, Pombo MA, Zhang P, et al iTAK: A Program for Genome-wide Prediction and Classification of Plant Transcription Factors, Transcriptional Regulators, and Protein Kinases. Mol Plant. 2016;9(12):1667–70. 10.1016/j.molp.2016.09.014. . 27717919

