# Supplementary material for: Prometheus, an omics portal for interkingdom comparative genomic analyses
Source: PLoS One. 2020 Oct 28;15(10):e0240191. doi: 10.1371/journal.pone.0240191 (PMC7592745; doi:10.1371/journal.pone.0240191)
Supplement: S1 File — (DOCX) [file pone.0240191.s001.docx]

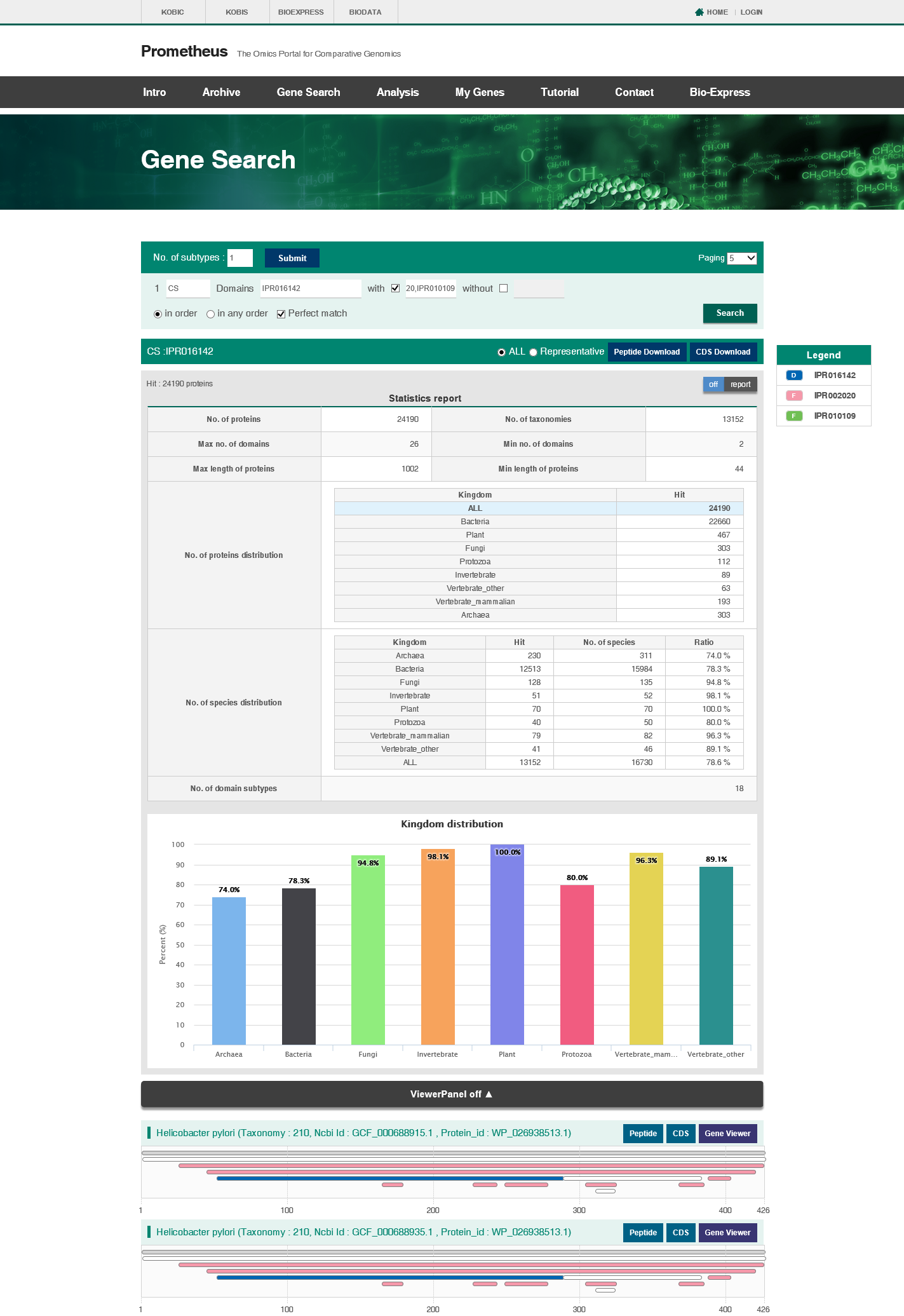


**Fig. S1.** Investigation of gene domain architectures in Gene Search.

The gene encoding citrate synthase in the TCA cycle was investigated. The numbers and ratios of citrate synthase genes detected in species are shown as a table and bar graph, respectively. The domain architectures of matched genes are shown in the bottom panel.


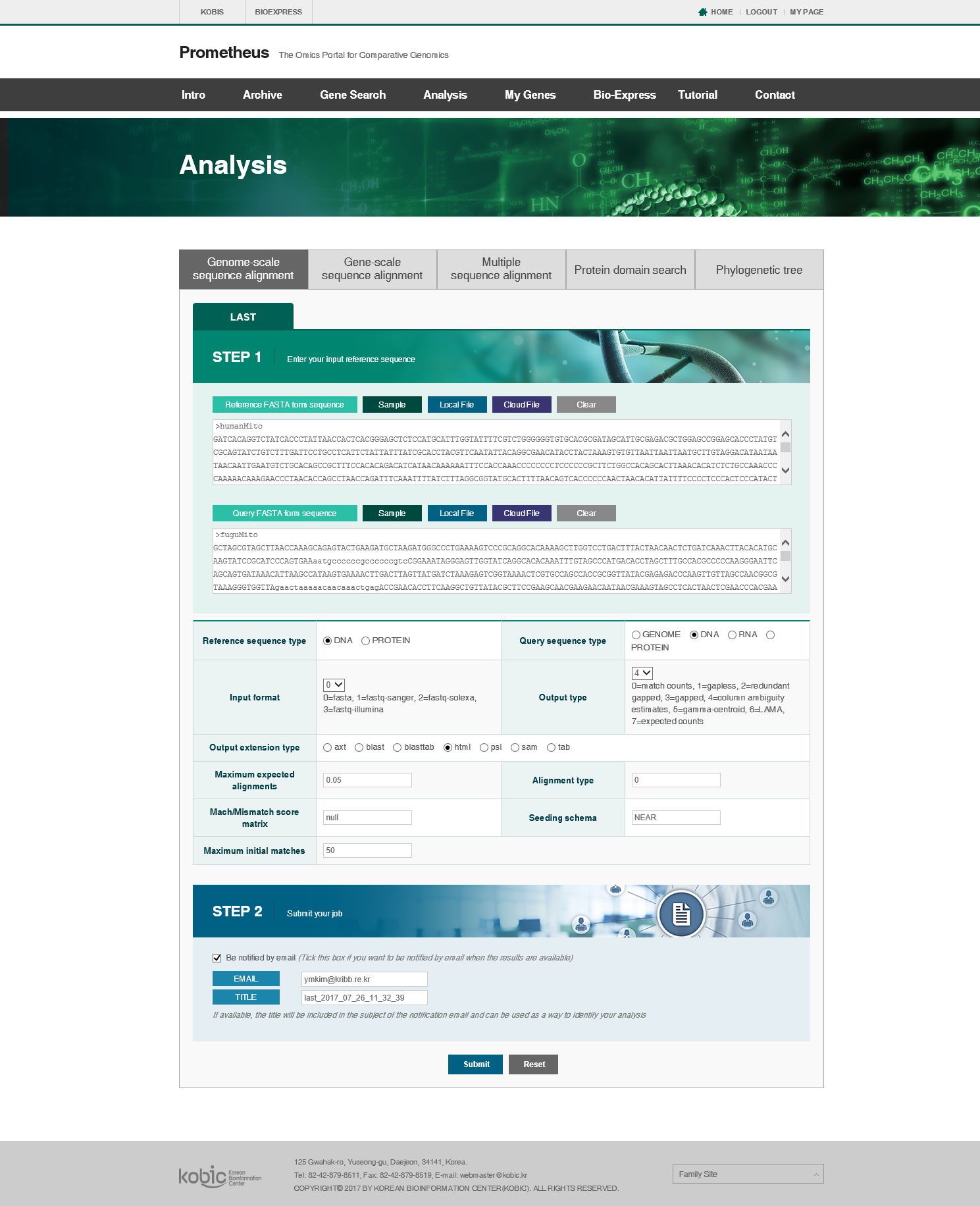


**Fig. S2.** Gene Analysis section in Prometheus.

Prometheus provides five bioinformatics analysis tools in the Gene Analysis section. Users can select each bioinformatics analysis tool by clicking on tabs. The sample data and default options are also provided for each tool.


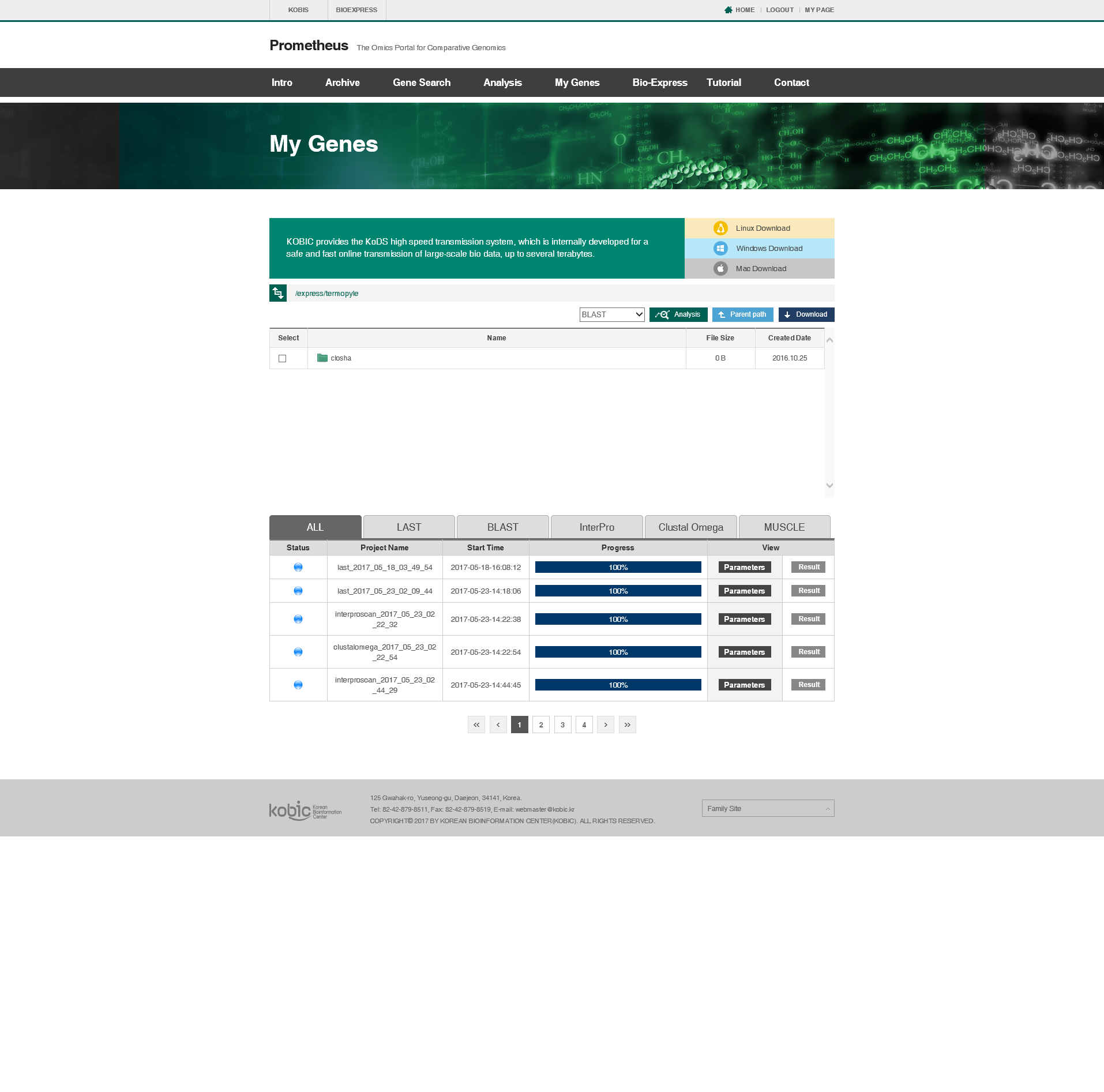


**Fig. S3.** My Genes in Prometheus.

Users can upload personal data or downloaded data from Prometheus and analyze them (upper panel). Users can monitor the options to run and the progress of analysis programs (lower panel).


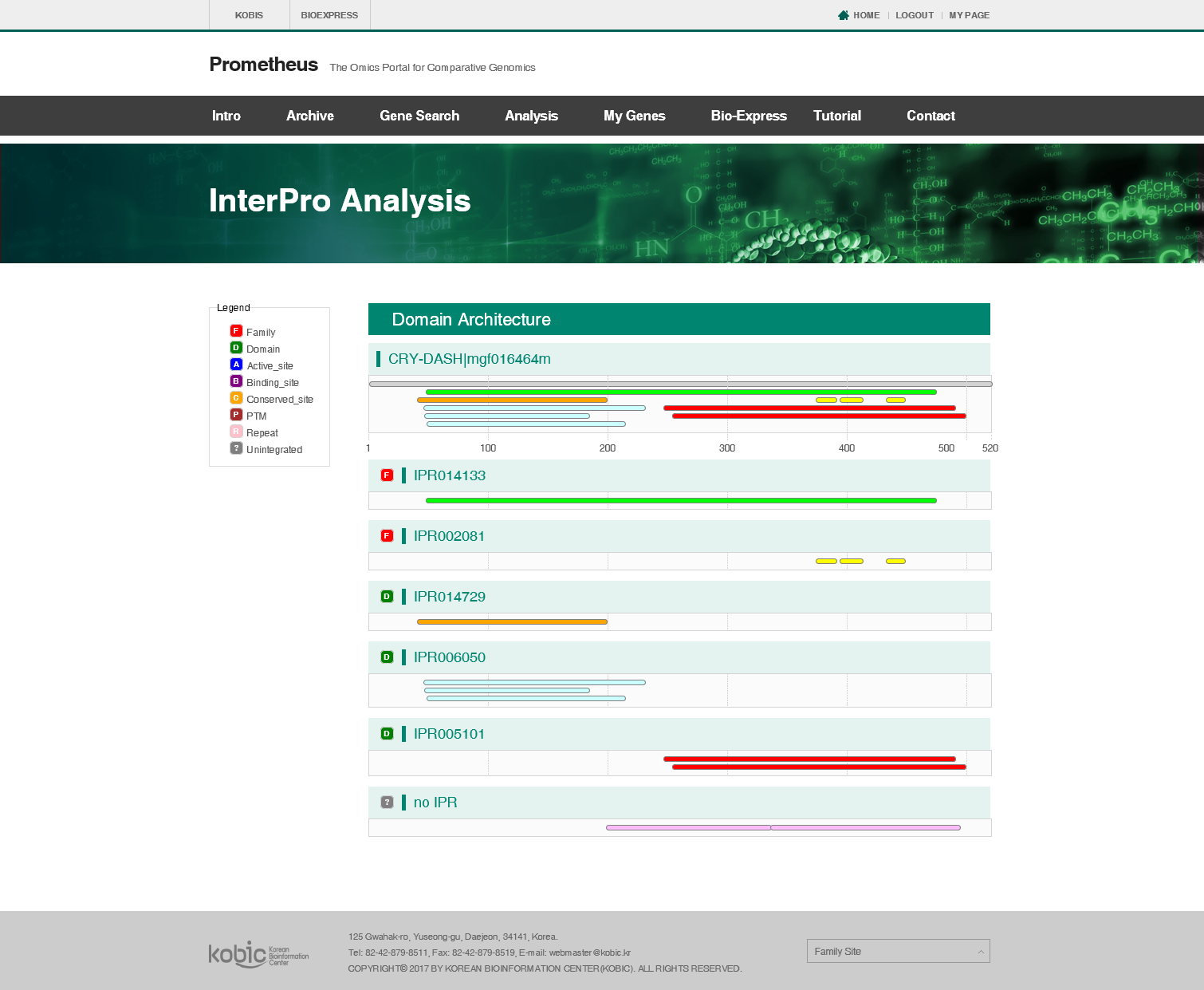


**Fig. S4.** InterPro result in Prometheus.

Results from InterProScan are provided in a tsv file format and domain architectures of genes of interest are also visualized as bars.


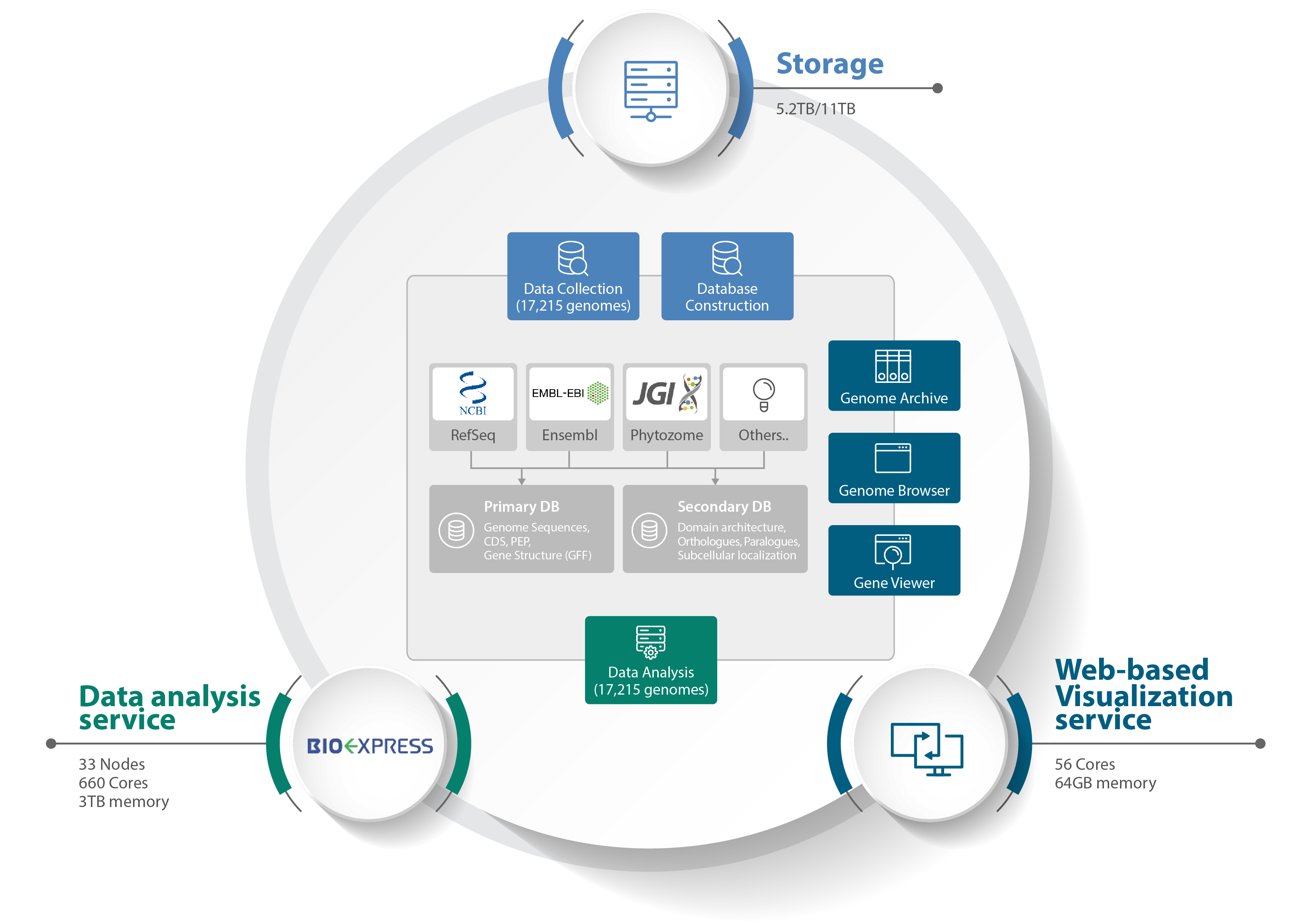


**Fig. S5. Infrastructure of Prometheus.**

Prometheus systems consist with three major parts, Data analysis service, Web-based Visualization service and Storage. All functions of Prometheus are run in the Bio-Express system, which consists of cluster nodes for bioinformatics analysis, HDFS storage for data deposition, cache solutions, and a distributed task scheduler. The Bio-Express hardware system consists of 40 Nodes, 900 core CPUs, 9.1 TB of memory, and 1.5 PB of disk storage in total.


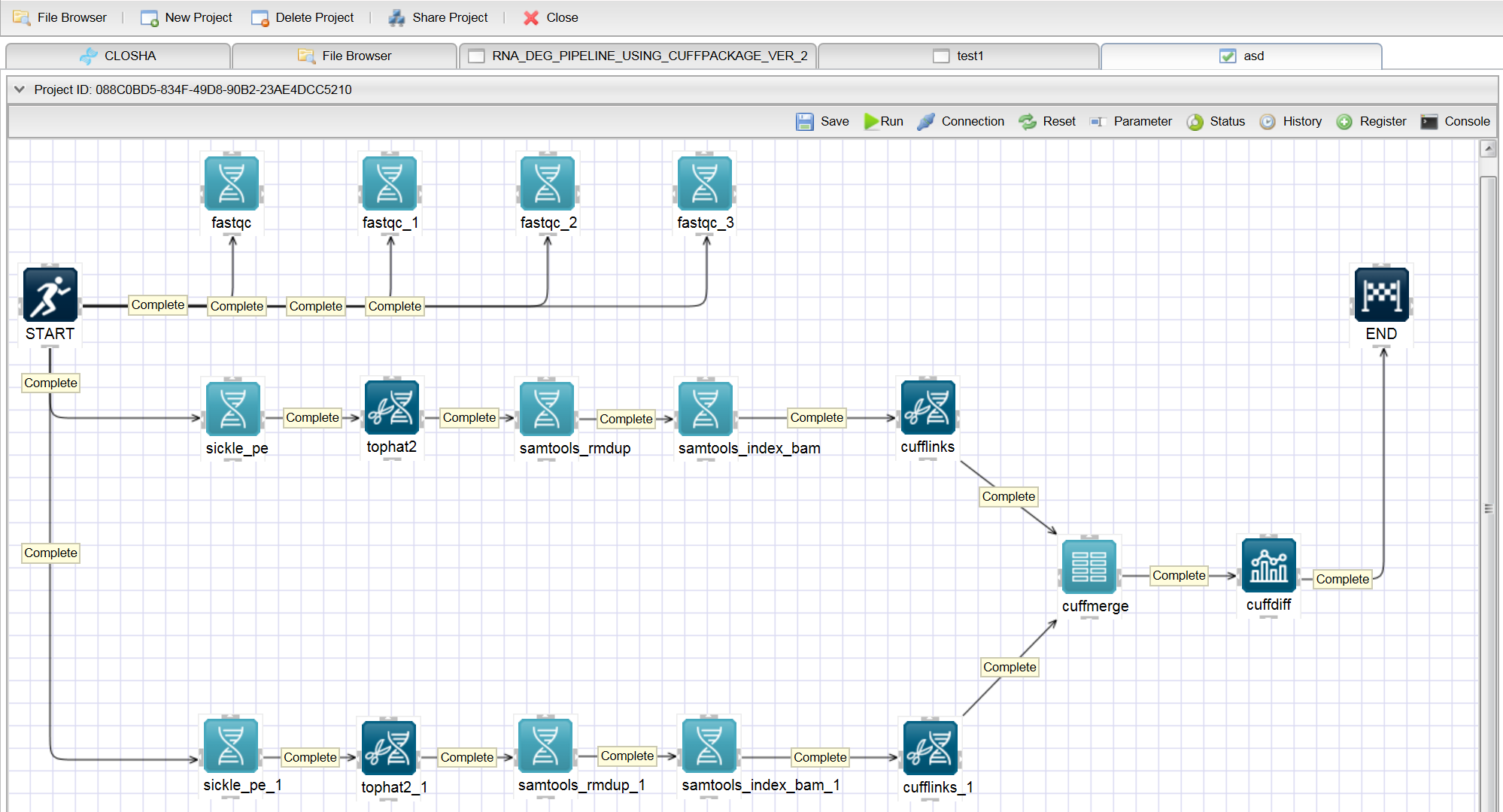


**Fig. S6.** Transcriptome analysis pipeline in Bio-Express.

Each program or module for transcriptome analysis is shown as an icon and users can construct their own analysis pipeline by linking each icon with arrows.


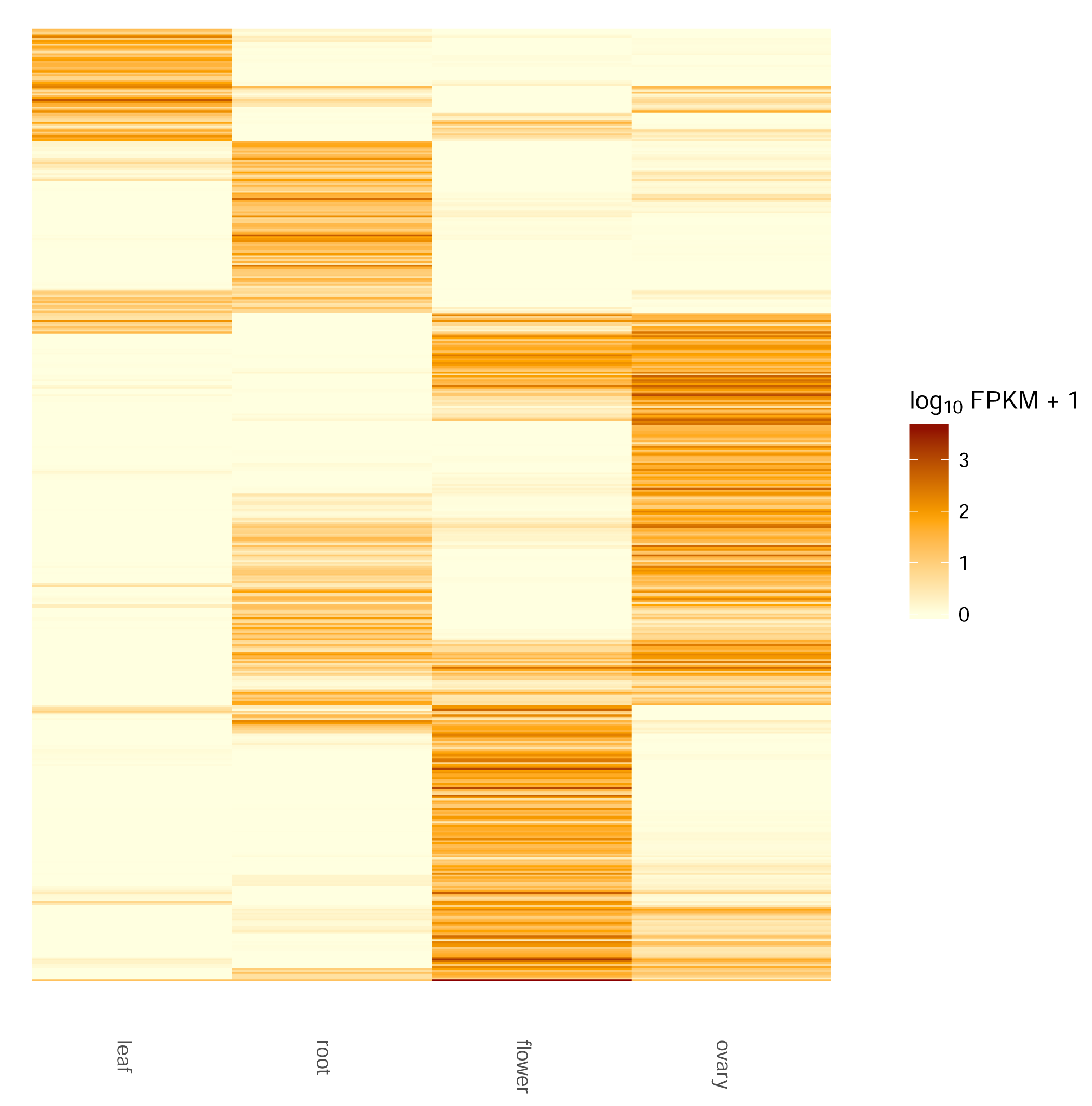


**Fig. S7.** Differentially expressed genes from the *Hibiscus syriacus* genome in four tissue types.

These differentially expressed genes were identified using the *H. syriacus* genome in Prometheus and TopHat and Cufflink programs in the Bio-Express platform.

| **Table S1.** List of species in Prometheus. | |  |
| --- | --- | --- |
| **Kingdom** | **No. of Species** | **No. of Versions** |
| Archaea | 311 | 327 |
| Bacteria | 15,984 | 16,358 |
| Fungi | 135 | 135 |
| Invertebrates | 52 | 55 |
| Plants | 70 | 104 |
| Protozoa | 50 | 50 |
| Vertebrates_mammalian | 82 | 123 |
| Vertebrates_other | 46 | 63 |
| **Total** | **16,730** | **17,215** |

**Table S2.** Statistics of primary database.

| **Source DB** | **Kingdom** | **No. of Species** | **Total** | **Ratio (%)** | **Version** | | **No. of Genes** | | **No. of CDS** | **No. of Protein** | |
| --- | --- | --- | --- | --- | --- | --- | --- | --- | --- | --- | --- |
| Ensembl | Fungi | 1 | 135 | 0.7 | 1 | | 7,126 | | 6,692 | 6,692 | |
|  | Invertebrates | 4 | 52 | 7.7 | 4 | | 93,641 | | 98,758 | 98,758 | |
|  | Vertebrates_mammalian | 43 | 82 | 52.4 | 43 | | 1,110,356 | | 1,008,078 | 1,008,078 | |
|  | Vertebrates_other | 21 | 46 | 45.7 | 21 | | 429,510 | | 495,087 | 495,087 | |
| Subtotal | | 69 |  | | | 69 | | 1,640,633 | 1,608,615 | | 1,608,615 |
| RefSeq | Archaea | 311 | 311 | 100.0 | 327 | | 867,420 | | 837,691 | 837,691 | |
|  | Bacteria | 15,984 | 15,984 | 100.0 | 16,358 | | 60,031,561 | | 57,913,073 | 57,898,444 | |
|  | Fungi | 134 | 135 | 99.3 | 134 | | 1,291,950 | | 1,270,035 | 1,269,916 | |
|  | Invertebrates | 51 | 52 | 98.1 | 51 | | 879,186 | | 890,278 | 890,258 | |
|  | Plants | 44 | 70 | 62.9 | 45 | | 1,202,462 | | 1,276,569 | 1,276,555 | |
|  | Protozoa | 50 | 50 | 100.0 | 50 | | 626,280 | | 614,278 | 614,278 | |
|  | Vertebrates_mammalian | 74 | 82 | 90.2 | 82 | | 2,363,247 | | 2,577,823 | 2,577,736 | |
|  | Vertebrates_other | 42 | 46 | 91.3 | 42 | | 888,488 | | 1,091,275 | 1,091,243 | |
| Subtotal | | 16,690 |  | | | 17,089 | | 68,150,594 | 66,471,022 | | 66,456,121 |
| Solgenomics | Plants | 7 | 70 | 10.0 | 11 | | 671,018 | | 730,821 | 730,821 | |
| TheOthers | Plants | 5 | 70 | 7.1 | 5 | | 187,585 | | 187,585 | 187,585 | |
| Phytozome | Plants | 43 | 70 | 61.4 | 43 | | 1,382,908 | | 1,732,263 | 1,732,263 | |
| **Total** | | **16,730** | **16,730** |  | **17,215** | | **72,032,738** | | **70,730,306** | **70,715,405** | |

**Table S3.** Statistics of secondary database from InterProScan.

| **Source DB** | **Kingdom** | **No. of Species** | **Total** | **Ratio (%)** | **No. of Versions** | **No. of Records** |
| --- | --- | --- | --- | --- | --- | --- |
| Ensembl | Fungi | 1 | 135 | 0.7 | 1 | 45,218 |
|  | Invertebrates | 4 | 52 | 7.7 | 4 | 1,015,257 |
|  | Vertebrates_mammalian | 43 | 82 | 52.4 | 43 | 12,985,696 |
|  | Vertebrates_other | 21 | 46 | 45.7 | 21 | 6,824,700 |
| Subtotal | | 69 |  | | 69 | 20,870,871 |
| Phytozome | Plants | 43 | 70 | 61.4 | 43 | 12,066,517 |
| RefSeq | Archaea | 311 | 311 | 100.0 | 327 | 4,536,692 |
|  | Bacteria | 15,984 | 15,984 | 100.0 | 16,358 | 384,234,199 |
|  | Fungi | 134 | 135 | 99.3 | 134 | 7,611,463 |
|  | Invertebrates | 51 | 52 | 98.1 | 51 | 8,067,628 |
|  | Plants | 44 | 70 | 62.9 | 45 | 9,435,494 |
|  | Protozoa | 50 | 50 | 100.0 | 50 | 3,350,146 |
|  | Vertebrates_mammalian | 74 | 82 | 90.2 | 82 | 36,394,680 |
|  | Vertebrates_other | 42 | 46 | 91.3 | 42 | 15,547,006 |
| Subtotal | | 16,690 |  | | 17,089 | 469,177,308 |
| Solgenomics | Plants | 7 | 70 | 10.0 | 11 | 5,037,832 |
| TheOthers | Plants | 5 | 70 | 7.1 | 5 | 1,130,404 |
| **Total** | | **16,730** | **16,730** |  | **17,215** | **508,282,932** |

**Table S4.** Statistics of secondary database from OrthoMCL.

| **Source DB** | **Kingdom** | **No. of Species** | **Total** | **Ratio (%)** | **No. of Records** | |
| --- | --- | --- | --- | --- | --- | --- |
| Ortholog | Fungi | 92 | 135 | 68 | 3,762,390 | |
|  | Invertebrates | 26 | 52 | 50 | 4,656,168 | |
|  | Plants | 51 | 70 | 73 | 92,774,828 | |
|  | Protozoa | 25 | 50 | 50 | 800,132 | |
|  | Vertebrates_mammalian | 42 | 82 | 51 | 38,055,626 | |
|  | Vertebrates_other | 16 | 46 | 35 | 8,515,110 | |
| Subtotal | | 252 |  | | 148,564,254 | |
| Paralog | Fungi | 92 | 135 | 68 | 1,049,784 | |
|  | Invertebrates | 26 | 52 | 50 | 1,257,416 | |
|  | Plants | 51 | 70 | 73 | 21,371,160 | |
|  | Protozoa | 25 | 50 | 50 | 1,601,092 | |
|  | Vertebrates_mammalian | 42 | 82 | 51 | 9,738,960 | |
|  | Vertebrates_other | 16 | 46 | 35 | 2,587,422 | |
| Subtotal | | 252 |  | | 37,605,834 | |
| **Total** | |  | | | | **186,170,088** |

**Table S5.** Statistics of secondary database from subcelluar localization programs.

| **Source DB** | **Kingdom** | **No. of Species** | **Total** | **Ratio (%)** | **No. of Records** | | |  |  |
| --- | --- | --- | --- | --- | --- | --- | --- | --- | --- |
| Multiloc2 | Fungi | 135 | 135 | 100 | 1,276,431 | | |  |  |
|  | Invertebrates | 52 | 52 | 100 | 988,338 | | |  |  |
|  | Plants | 70 | 70 | 100 | 3,656,331 | | |  |  |
|  | Protozoa | 50 | 50 | 100 | 613,972 | | |  |  |
|  | Vertebrates_mammalian | 82 | 82 | 100 | 3,583,474 | | |  |  |
|  | Vertebrates_other | 46 | 46 | 100 | 1,585,924 | | |  |  |
| Subtotal | | 435 |  | | | 11,704,470 | | |  |
| TargetP | Fungi | 135 | 135 | 100 | 1,276,608 | | |  |  |
|  | Invertebrate | 52 | 52 | 100 | 989,016 | | |  |  |
|  | Plants | 70 | 70 | 100 | 3,658,071 | | |  |  |
|  | Protozoa | 50 | 50 | 100 | 614,278 | | |  |  |
|  | Vertebrates_mammalian | 82 | 82 | 100 | 3,585,814 | | |  |  |
|  | Vertebrates_other | 46 | 46 | 100 | 1,586,330 | | |  |  |
| Subtotal | | 435 |  | | | 11,710,117 | | |  |
| TMHMM2 | Archaea | 311 | 311 | 100 | 837,691 | | |  |  |
|  | Bacteria | 15,835 | 15,984 | 99 | 57,450,117 | | |  |  |
|  | Fungi | 135 | 135 | 100 | 1,276,608 | | |  |  |
|  | Invertebrates | 52 | 52 | 100 | 989,016 | | |  |  |
|  | Plants | 70 | 70 | 100 | 3,658,071 | | |  |  |
|  | Protozoa | 50 | 50 | 100 | 614,278 | | |  |  |
|  | Vertebrates_mammalian | 82 | 82 | 100 | 3,585,814 | | |  |  |
|  | Vertebrates_other | 46 | 46 | 100 | 1,586,330 | | |  |  |
| Subtotal | | 16,581 |  | | | 69,997,925 | | |  |
| **Total** | |  | | | | | **93,412,512** | | |

**Table S6.** Comparison of other comparative portals.

|  | CoGe | Ensembl | PLAZA 4.0 | MicobesOnline | Prometheus |
| --- | --- | --- | --- | --- | --- |
| Genomes | 49,694 | 91,100 | 84 | 1,957 | 17,215 |
| -Bacteria | - | 90,000 | - | 1,752 | 16,358 |
| -Archaea | - | - | - | 94 | 327 |
| -Protozoa | - | 22 | - | 7 | 50 |
| -Fungi | - | 811 | - | 99 | 135 |
| -Plants | - | 61 | 84 | 5 | 104 |
| -Animals | - | 206 | - | - | 241 |
| Gene/Protein features | Gene | YES | YES | YES | YES |
| Orthologue/Paralogue | YES | YES | YES | YES | YES |
| Protein domain | NO | YES | YES | YES | YES |
| Domain architecture-based gene search | NO | NO | NO | NO | YES |
| Subcellular localization | NO | YES | NO | NO | YES |

**Table S7**. Numbers of unique proteins in Prometheus.

| Organismal divisions | Databases | Numbers of unique proteins | Total |
| --- | --- | --- | --- |
| Archaea | Refseq | 659,856 | 659,856 |
| Bacteria | Refseq | 50,801,861 | 50,801,861 |
| Fungi | Refseq | 970,448 | 970,448 |
| Invertebrates | Ensembl | 148,766 | 960,353 |
|  | Refseq | 811,587 |  |
| Plants | Refseq | 1,934,415 | 5,133,536 |
|  | Phytozome | 2,336,072 |  |
|  | Solgenomics | 696,270 |  |
|  | The others | 166,779 |  |
| Protozoa | Refseq | 448,210 | 448,210 |
| Vertebrates_mammalian | Ensembl | 1,814,805 | 5,673,782 |
|  | Refseq | 3,858,977 |  |
| Vertebrates_other | Ensembl | 882,873 | 2,417,426 |
|  | Refseq | 1,534,553 |  |
| Total |  | 67,065,472 | 67,065,472 |

**Table S8.** Performance of Gene Search.

| Domains | Proteins | Run-time(duration) |
| --- | --- | --- |
| IPR013654,IPR000014,IPR003018,IPR013515,IPR000014,IPR013767,IPR000014,IPR013767,IPR003661,IPR003594 | 458 | <10 sec |
| IPR014001,IPR011545,IPR001650,IPR005034,IPR003100,IPR000999,IPR000999,IPR014720,IPR014720 | 472 | <10 sec |
| IPR003349,IPR003347,IPR004198,IPR003888,IPR003889 | 397 | <7 sec |
| IPR004330,IPR018289,IPR006564,IPR007527 | 247 | <5 sec |
| IPR006050,IPR005101,IPR020978 | 227 | <5 sec |

**Table S9.** Validation results of transcriptional factors using Gene Search in Prometheus.

| **TF** | **Domain Architecture** | **Family/Site** | **Prometheus** | **iTAK** | **Accuracy (%)** |
| --- | --- | --- | --- | --- | --- |
| FAR1 | IPR004330,IPR018289,IPR007527,IPR006564 | IPR031052 | 4,758 | 4,700 | 98.78 |
| MADS | IPR002100 | IPR002487 | 4,449 | 4,425 | 99.46 |
| NAC | IPR003441 | - | 12,007 | 11,606 | 96.66 |
| B3-Type TF | IPR015300,IPR003340 | - | 9,078 | 8,590 | 94.62 |
| Homeobox | IPR009057,IPR001356 | IPR000047,IPR017970 | 6,406 | 6,405 | 99.98 |
| GARP-ARR-B | IPR011006,IPR009057 | IPR017053 | 735 | 692 | 94.15 |
| AUX-IAA | IPR033389,IPR000270 | IPR003311 | 2,218 | 2,190 | 98.74 |
| C2C2-Dof | IPR003851 | - | 3,518 | 3,516 | 99.94 |
| C2C2-CO | IPR000315,IPR010402 | - | 1,450 | 1,448 | 99.86 |
| C2C2-GATA | IPR010399,IPR010402 | - | 648 | 608 | 93.83 |
| CAMTA (Plant) | IPR005559,IPR002909,IPR020683 | IPR002110,IPR000048 | 413 | 376 | 91.04 |
| CCAAT | IPR009072,IPR003958 | - | 6,137 | 5,733 | 93.42 |
| BES1 | IPR008540 | - | 1,028 | 1,027 | 99.90 |
| LIM | IPR001781 | IPR022087, IPR003903 | 365 | 314 | 86.03 |

**Table S10.** Gene Search results of TCA cycle genes.

| **Genes** | **Query IPR terms for Gene Search** | | **No. (%) of sequences identified** | | | | | | |
| --- | --- | --- | --- | --- | --- | --- | --- | --- | --- |
|  |  |  | **Bacteria** | **Plant** | **Fungi** | **Protozoa** | **Invertebrate** | **Vertebrate** | **Archaea** |
|  |  |  | ***n*=15,984** | ***n*=70** | ***n*=135** | ***n*=50** | ***n*=52** | ***n*=128** | ***n*=311** |
| CS | Domain | IPR016142 | 12513 (78.3) | 70 (100) | 128 (94.8) | 40 (80.0) | 51 (98.1) | 120 (93.8) | 230 (74.0) |
|  | With | IPR002020,IPR010109 |  |  |  |  |  |  |  |
| ACO1 | Domain | IPR001030,IPR015928 | 10844 (67.8) | 69 (98.6) | 126 (93.3) | 40 (80.0) | 51 (98.1) | 125 (97.7) | 186 (59.8) |
|  | With | IPR015931,IPR015932,IPR015937,IPR006249,IPR029784 |  |  |  |  |  |  |  |
| ACO2 | Domain | IPR001030,IPR015932 | 13091 (81.9) | 70 (100) | 127 (94.1) | 40 (80.0) | 51 (98.1) | 126 (98.4) | 278 (89.4) |
|  | With | IPR015931,IPR015928,IPR000573,IPR015937,IPR006248 |  |  |  |  |  |  |  |
| IDH1 | Domain | IPR024084 | 2463 (15.4) | 70 (100) | 124 (91.9) | 40 (80.0) | 50 (96.2) | 127 (99.2) | 1 (0.3) |
|  | With | IPR004790 |  |  |  |  |  |  |  |
| IDH2 | Domain | IPR024084 | 2463 (15.4) | 70 (100) | 124 (91.9) | 40 (80.0) | 50 (96.2) | 127 (99.2) | 1 (0.3) |
|  | With | IPR004790 |  |  |  |  |  |  |  |
| IDH3A | Domain | IPR024084 | 13448 (84.1) | 70 (100) | 128 (94.8) | 27 (54.0) | 52 (100.0) | 127 (99.2) | 279 (89.7) |
|  | With | IPR001804,IPR004434 |  |  |  |  |  |  |  |
| IDH3B | Domain | IPR024084 | 13448 (84.1) | 70 (100) | 128 (94.8) | 27 (54.0) | 52 (100.0) | 127 (99.2) | 279 (89.7) |
|  | With | IPR001804,IPR004434 |  |  |  |  |  |  |  |
| IDH3G | Domain | IPR024084 | 13448 (84.1) | 70 (100) | 128 (94.8) | 27 (54.0) | 52 (100.0) | 127 (99.2) | 279 (89.7) |
|  | With | IPR001804,IPR004434 |  |  |  |  |  |  |  |
| OGDH-1 | Domain | IPR029061 | 12462 (78.0) | 70 (100) | 133 (98.5) | 41 (82.0) | 52 (100.0) | 127 (99.2) | 149 (47.9) |
|  | With | IPR032106,IPR001017,IPR011603 |  |  |  |  |  |  |  |
| DLST | Domain | IPR000089,IPR023213 | 415 (2.6) | 64 (91.4) | 122 (90.4) | 36 (72.0) | 47 (90.4) | 124 (96.9) | 1 (0.3) |
|  | With | IPR011053,IPR001078,IPR006255 |  |  |  |  |  |  |  |
| DLD | Domain | IPR023753 | 15012 (93.9) | 70 (100) | 128 (94.8) | 45 (90.0) | 52 (100.0) | 127 (99.2) | 286 (92.0) |
|  | With | IPR016156,IPR004099,IPR006258 |  |  |  |  |  |  |  |
| SUCLG1 | Domain | IPR016040,IPR003781,IPR016102,IPR005811 | 10871 (68.0) | 61 (87.1) | 124 (91.9) | 30 (60.0) | 47 (90.4) | 122 (95.3) | 209 (67.2) |
|  | With | IPR005810 |  |  |  |  |  |  |  |
| SUCLG2 | Domain | IPR013650,IPR013815,IPR013816,IPR016102,IPR005811 | 10968 (68.6) | 66 (94.3) | 118 (87.4) | 38 (76.0) | 51 (98.1) | 122 (95.3) | 218 (70.1) |
|  | With | IPR005809 |  |  |  |  |  |  |  |
| SUCLA2 | Domain | IPR011761,IPR016102 | 10460 (65.4) | 56 (80.0) | 75 (55.6) | 10 (20.0) | 36 (69.2) | 98 (76.6) | 248 (79.7) |
|  | With | IPR013650,IPR013815,IPR013816,IPR005811,IPR005809 |  |  |  |  |  |  |  |
| SdhA-1 | Domain | IPR027477,IPR015939 | 9899 (61.9) | 65 (4.4) | 6 (4.4) | 10 (20.0) | 7 (13.5) | 30 (23.4) | 203 (83.9) |
|  | With | IPR023753,IPR015939,IPR014006,IPR011281 |  |  |  |  |  |  |  |
| SdhA-2 | Domain | IPR023753,IPR027477 | 12345 (77.2) | 68 (97.1) | 124 (91.9) | 37 (74.0) | 50 (96.2) | 120 (93.8) | 261 (83.9) |
|  | With | IPR023753,IPR027477,IPR030664,IPR014006,IPR011281 |  |  |  |  |  |  |  |
| SdhB | Domain | IPR012675,IPR001041,IPR025192,IPR009051,IPR017896 | 8832 (55.3) | 61 (87.1) | 118 (87.4) | 28 (56.0) | 48 (92.3) | 113 (88.3) | 178 (57.2) |
|  | With | IPR004489 |  |  |  |  |  |  |  |
| FH | Domain | IPR022761,IPR018951 | 12381 (77.5) | 69 (98.6) | 125 (92.6) | 19 (38.0) | 48 (92.3) | 120 (93.8) | 172 (55.3) |
|  | With | IPR000362,IPR005677,IPR024083,IPR008948 |  |  |  |  |  |  |  |
| MDH1 | Domain | IPR016040,IPR001236,IPR015955,IPR022383 | 11813 (73.9) | 70 (100) | 114 (84.4) | 45 (90.0) | 52 (100.0) | 127 (99.2) | 148 (47.6) |
|  | With | IPR001557,IPR010945,IPR011274 |  |  |  |  |  |  |  |
| MDH2 | Domain | IPR016040,IPR001236,IPR015955,IPR022383 | 11805 (73.9) | 69 (98.6) | 114 (84.4) | 12 (24.0) | 20 (38.5) | 127 (99.2) | 148 (47.6) |
|  | With | IPR001557,IPR010097 |  |  |  |  |  |  |  |

**Table S11.** Gene Search results of photolyase/cryptochrome gene family.

| **Genes** | **Query IPR terms for Gene Search** | | **No. (%) of sequences identified** | | | | | | |
| --- | --- | --- | --- | --- | --- | --- | --- | --- | --- |
|  |  |  | **Bacteria** | **Plant** | **Fungi** | **Protozoa** | **Invertebrate** | **Vertebrate** | **Archaea** |
|  |  |  | ***n*=15984** | ***n=*70** | ***n=*135** | ***n=*50** | ***n=*52** | ***n=*128** | ***n=*311** |
| Animal-type | Domain | IPR006050,IPR005101 | 425 (2.7) | 68 (97.1) | 54 (40.0) | 14 (28.0) | 35 (67.3) | 127 (99.2) | 24 (7.7) |
|  | With |  |  |  |  |  |  |  |  |
|  | Without | IPR014133,IPR002081,IPR008148,IPR014134 |  |  |  |  |  |  |  |
| CPDI | Domain | IPR006050,IPR005101 | 8382 (52.4) | 67 (95.7) | 73 (54.1) | 11 (22.0) | 25 (48.1) | 39 (30.5) | 133 (42.8) |
|  | With | IPR002081 |  |  |  |  |  |  |  |
|  | Without |  |  |  |  |  |  |  |  |
| CPDII | Domain | IPR006050,IPR005101 | 171 (1.1) | 66 (94.3) | 1 (0.7) | 19 (38.0) | 35 (67.3) | 45 (35.2) | 12 (3.9) |
|  | With | IPR008148 |  |  |  |  |  |  |  |
|  | Without |  |  |  |  |  |  |  |  |
| CRY-DASH | Domain | IPR006050,IPR005101 | 891 (5.6) | 61 (87.1) | 44 (32.6) | 3 (6.0) | 6 (11.5) | 37 (28.9) | 22 (7.1) |
|  | With | IPR014133 |  |  |  |  |  |  |  |
|  | Without |  |  |  |  |  |  |  |  |
| Plant-type | Domain | IPR006050,IPR005101 | - | 58 (82.9) | - | - | - | - | - |
|  | With | IPR014134 |  |  |  |  |  |  |  |
|  | Without |  |  |  |  |  |  |  |  |
| Plant-type Cry1 | Domain | IPR014729,IPR005101,IPR020978 | - | 52 (74.3) | - | - | - | - | - |
|  | With | IPR014134 |  |  |  |  |  |  |  |
|  | Without |  |  |  |  |  |  |  |  |
| Plant-type Cry2 | Domain | IPR006050,IPR005101 | - | 51 (72.9) | - | - | - | - | - |
|  | With | IPR014134 |  |  |  |  |  |  |  |
|  | Without | IPR020978 |  |  |  |  |  |  |  |

**Table S12**. Lists of programs provided by Bio-Express.

| Name of Pipeline | Description |
| --- | --- |
| CLUSTALO_PIPELINE_VER_1 | Clustao is a general purpose multiple sequence alignment program |
| EPIGENOME_ANALYSIS_PIPELINE_USING_HOMER_VER_2 | Pipelines for epigenome analysis using Homer (ver 2). |
| EXOME_ANALYSIS_PIPELINE_USING_GATK_VER_2 | Exome sequencing analysis pipeline using GATK (ver 2). |
| GSA_PIPELINE_VER_1 | Variant calling program using GATK best predict with Sanger sequencing. |
| HADOOP_BIG_BWA_MEM_PIPELINE_VER_1 | Burrows Wheeler Aligner (BWA) program for Hadoop system. |
| HADOOP_BLASTP_PIPELINE_VER_1 | Blastp program for Hadoop system. |
| INTERPROSCAN_PIPELINE_VER_1 | InterProScan program for Hadoop system. |
| METAGENOME_ANALYSIS_PIPELINE_USING_QIIME_VER_1 | Metagenome analysis program for taxonomic assignment or sequence alignment program. |
| MUSCLE_PIPELINE_VER_1 | MUSCLE program for Hadoop system |
| RNA_ANALYSIS_PIPELINE_USING_EMSAR_VER_1 | RNA analysis pipeline using EMSAR for Hadoop system. |
| VARIANT_CALLING_PIPELINE_USING_SNPEFF_VER_2 | Variant calling pipeline using SNPEFF (ver 2) for Hadoop system. |
| LAST_PIPELINE_VER_1 | LAST program for Hadoop system. |
| HADOOP_BAM_PIPELINE_VER_1 | Hadoop BAM is a Java library for the manipulation of files in common bioinformatics formats using the Hadoop MapReduce framework with the Picard SAM JDK and command line tools similar to SAMtools. |
| RNA_DEG_PIPELINE_USING_CUFFPACKAGE_VER_2 | RNA-Seq analysis pipelines using Cufflink package for DEG analysis. |
| RNA_DEG_PIPELINE_USING_VOOM_VER_2 | RNA-Seq analysis pipeline using voom for DEG analysis. |
| BOWTIE_ALIGNMENT_PIPELINE_VER2 | Bowtie analysis pipeline for Hadoop system. |
| RNASeq_TOPHAT2_CUFFLINKS_PIPELINE | RNA-Seq analysis pipelines using TopHat2 and Cufflink package for Hadoop system. |
| POSTECH_EPIGENOME_SEQUENCING_FASTQC_BOWTIE_MACS_PIPELINE | Pipelines for epigenome analysis using Bowtie and MACS. |
| RNASeq_KALLISTO_PIPELINE | This pipeline is an RNA sequencing pipeline that performs pseudo alignment and quantification quickly using the Kallisto program. |
| RNASeq_EMSAR_PPIPELINE | This pipeline Analyze the RNA-seq to get isoform level estimates by EMSAR and then it will give you gene level expression level estimates using isoform level estimates |
| RNASeq_STARFUSION_PIPELINE | Detection of fusion using STAR Fusion with RNA-seq. |
| RNASeq_STAR_RSEM_PIPELINE | This pipeline is an RNA sequencing pipeline that aligns with the STAR program and performs quntification with RSEM. |
| RNASeq_STAR_HTSEQ_PIPELINE | This pipeline is an RNA sequencing pipeline that aligns with the STAR program and performs quantification with HTSeq. |
| mtDNA_Workflow | This repository includes the new mtDNA-Server workflow starting with BAM files. The workflow can be executed locally without transferring any data. For initial read mapping (FASTQ to BAM), we recommend using bwa mem. |
| GATK_VARIANTS_CALLING_PIPELINE | GATK variants calling pipeline (v3.5) |
| DEGs_PIPELINE | DEG analysis pipeline by comparing expression value between tumor and normal group. |
| WHOLE_GENOME_SOMATIC_MUTATION_CALLING_PIPELINE_WITH_ANNOTATION | Somatic variants calling program using GATK4 with tumor and normal tissues. |
| RNASeq_HTSeq_VOOM_PIPELINE | RNA-seq analysis pipeline using Bowtie2 and TopHat2 for read alignment and Voom for DEG analysis. |

**Supplementary Note**

**Section 1: Construction of primary and secondary database**

The database consisted of primary and secondary data tables in the Prometheus was constructed using MySQL database management system. In database, primary data tables were created through data is opened in five public databases and secondary data tables were constructed by parsing results of bioinformatics tools such as InterProScan, OrthoMCL, MultiLoc2, and TargetP (Fig. S7).


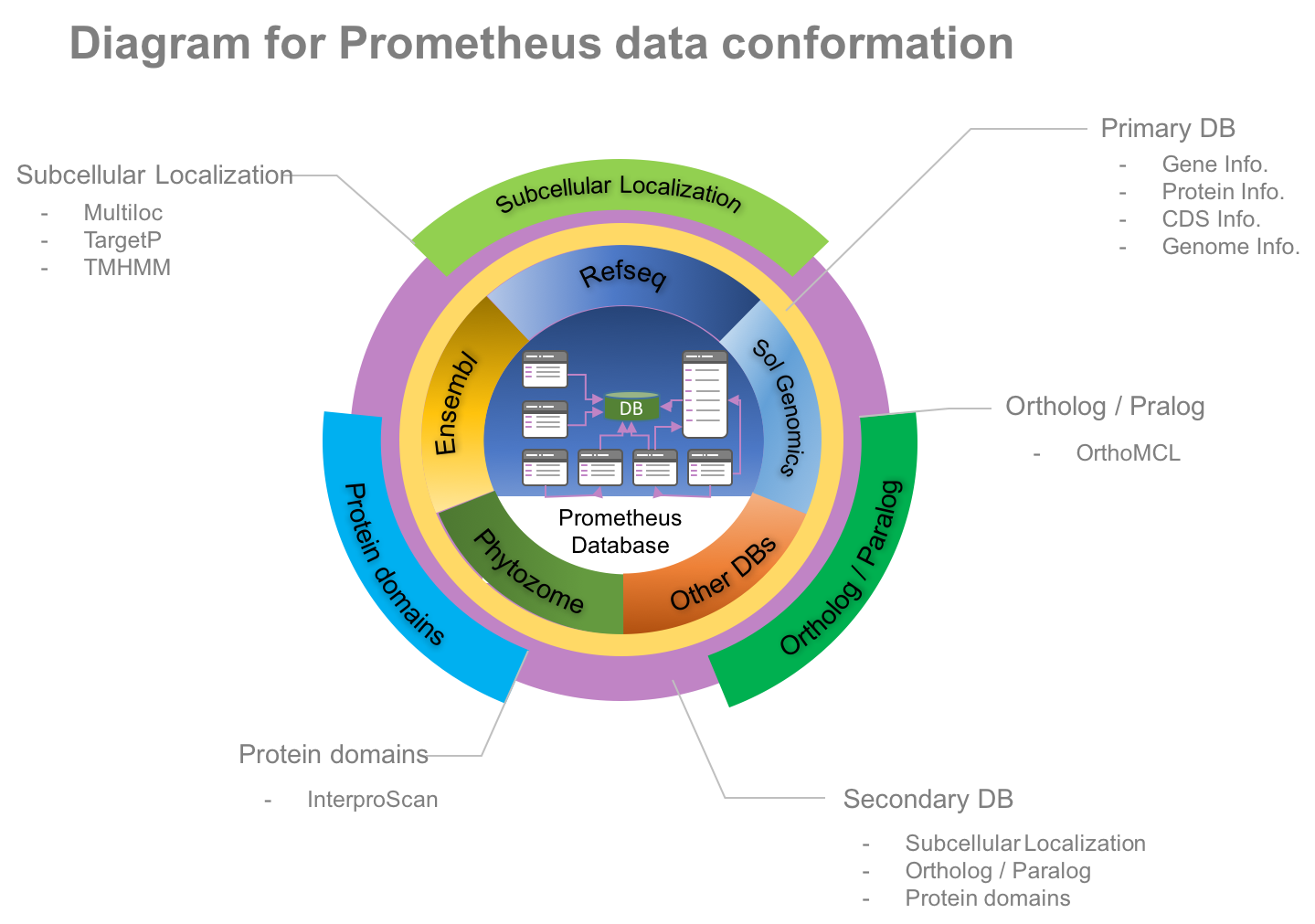


**Fig. S7**. Diagram for data conformation of Prometheus. Prometheus data architectures composed of primary (yellow circle) and secondary data (purple circle) and primary and secondary data were obtained from public database and bioinformatics analyses, respectively.

Primary data tables are constructed by genome information from public database such as RefSeq, Ensembl, Sol Genomics, and Phytozome. These tables contain information of gene features, CDSs, proteins, and meta-information of genome. These information are separated by kingdom and source databases by parsing data including gene features, CDSs, and proteins except genome sequence. Secondary data tables contain ortholog/paralog, subcellular localization, and domain architectures of proteins from various bioinformatics tools such as InterPro, OrthoMCL, MultiLoc2, and TargetP. These primary and secondary tables are connected by gene ID and user can efficiently find any information in the Prometheus using gene ID. Among data tables, some tables contain more than 100 million records and these tables may cause problems for retrieving and handling data. For example, InterPro data tables consisted of InterPro results contained more than 400 million records in a single table. Thus, partitioning and indexing techniques of MySQL were applied to the database and index data tables are created composed of frequently queried data to enhance performance of data search (Fig. S8).


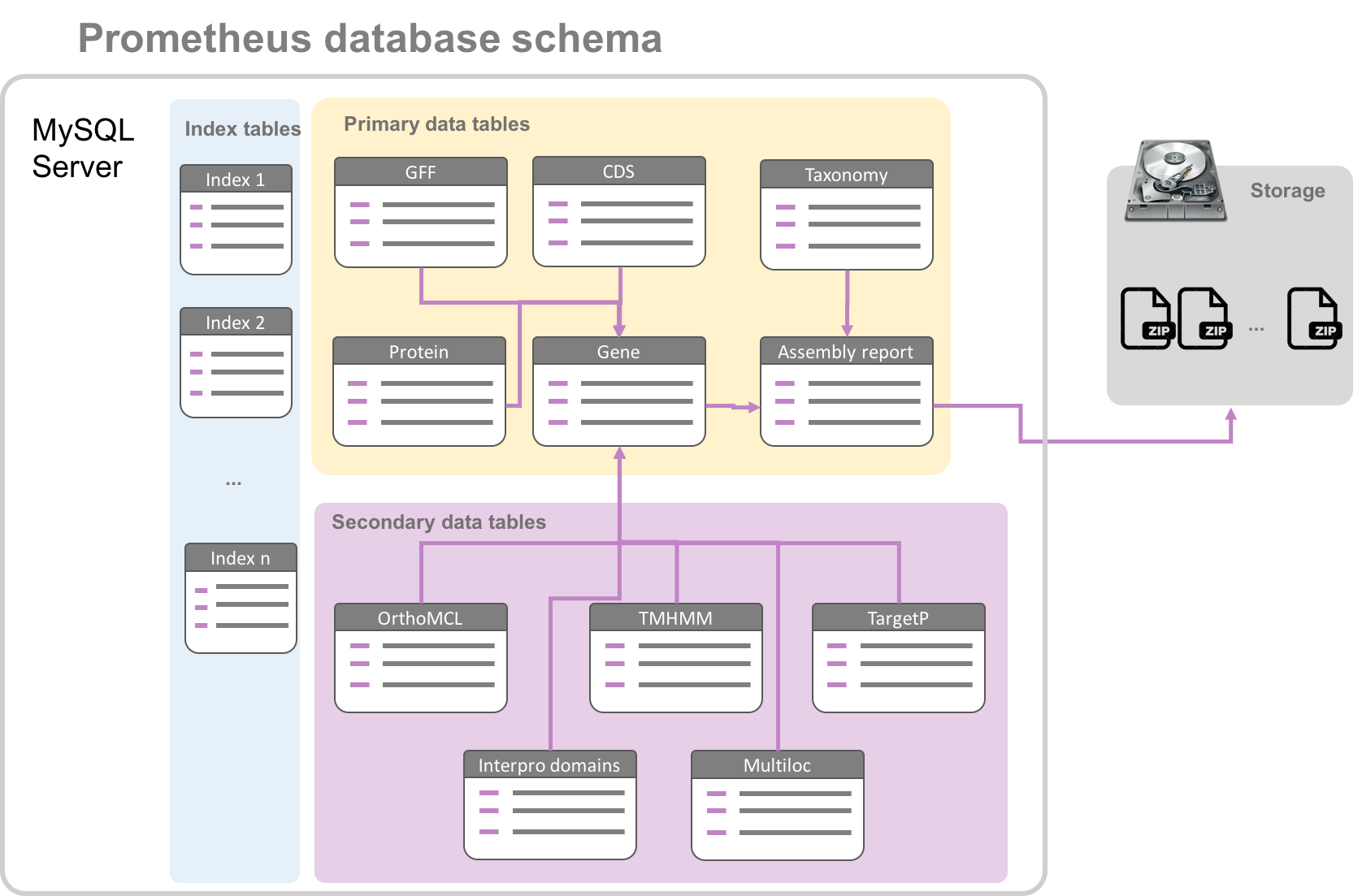


**Fig. S8**. Database schema of Prometheus. Database of Prometheus are consisted of three parts such as primary (yellow rectangle), secondary (purple rectangle), and index tables (blue rectangle).

**Section 2: Subcellular localization Analyses**

**Protein subcellular localization prediction**

In cell biology, lipid bilayer membrane has important rules. Biochemical processes such as lipid catabolism, oxidative phosphorylation, and photosynthesis are occurred in or on membrane surface or require a membrane-bound protein to transport of substance such as hydrogen ion. In the cell, there are many types of organelles, which have their own specific functions with exquisite mechanisms that consist of various types of proteins. In those organelles, proteins make complex local specific machinery and work like an interlocking gear or a well harmonized orchestra. Proteins function differently by location. Thus, we can conjecture function of protein based on the location of the protein. Hence, the information of protein localization is important to understand the function of the protein.

To provide the information of protein subcellular localization in database, we used three programs which predict the subcellular localization from protein sequence data: TMHMM [1], TargetP [2] and MultiLoc2 [3].

**TMHMM**

For the program TMHMM, its name stands for transmembrane hidden Markov model, which allows to predict the location and orientation of alpha helices in membrane-spanning proteins. The model reflects the overall topology of the transmembrane protein, which is cyclic with states of cytoplasmic side (a loop on the cytoplasmic side and a globular domain), membrane (helix cap on the cytoplasmic side, helix core and helix cap on the non-cytoplasmic side), and non-cytoplasmic side (short or long loop for the non-cytoplasmic side and globular domain). In addition to the location of a transmembrane helix, it gives knowledge whether functional part of protein faces inwards or outwards and thus information for making functional inference can be provided.

TMHMM version 2.0c was used to predict transmembrane helices of protein sequences in all organismal categories (archaea, bacteria, fungi, invertebrate, plant, protozoa, vertebrate mammalian, and vertebrate other) with default option. A total of 66,007,794 protein sequences were processed.

**TargetP**

TargetP is a neural network based predictor designed to assign specific localization for the mitochondrion, the chloroplast, the secretory pathway (ER/Golgi apparatus/secreted) and ‘other’ to a query protein in eukaryote using N-terminal sequence information. TargetP has two layers of neural networks, where the first one includes three independent networks for three types of N-terminal targeting sequence: mitochondrial targeting peptides (mTPs), chloroplast transit peptides (cTPs) and signal peptides (SPs) respectively, and the second is an integrator that makes the actual prediction of four different localizations using outcome of the first layer. TargetP is an integrative and extensive work based on SignalP [4] and ChloroP [5], that were designed to identify SPs and cTPs respectively.

TargetP version 1.1b was used to predict four localizations of proteins in eukaryotic categories (fungi, invertebrate, plant, protozoa, vertebrate mammalian, and vertebrate other) and SignalP version 4.1c and ChloroP version 1.1 was used to predict cleavage sites for SPs and cTPs respectively. Eukaryotic organisms were discriminated between plant and non-plant, and cTPs were predicted for plant only. A total of 7,719,986 protein sequences were processed.

**MultiLoc2**

As a support vector machine (SVM) based predictor, MultiLoc2 (MultiLoc2-HighRes) can cover all 11 main eukaryotic subcellular localizations: nuclear, cytoplasmic, mitochondrial, chloroplast, extracellular, plasma membrane, peroxisomal, endoplasmic reticulum, Golgi apparatus, lysosomal, and vacuolar localizations. By MultiLoc2, probability estimates for each localization is produced. For this prediction, MultiLoc2 combines the output of six specialized subpredictors: SVMTarget, SVMSA, SVMaac, MotifSearch, GOLoc, and PhyloLoc. Brief descriptions of these subpredictors are as follows: 1) SVMTarget is for recognizing N-terminal targeting sequences (SPs, mTPs, and cTPs). 2) SVMSA is for recognizing signal anchors which can be present in membrane proteins of the secretory pathway instead of a signal peptide. 3) SVMaac is for analyzing the overall amino acid composition. 4) MotifSearch is for recognizing relevant sequence motifs like nuclear localization signals (NLSs), Lys-Asp-Glu-Leu target peptide sequences (KDELs), Ser-Lys-Leu target peptide sequence (SKLs), or DNA binding domains. 5) GOLoc is for analyzing Gene Ontology terms derived from the protein sequence. 6) PhyloLoc is for analyzing the phylogenetic profiles of the query sequences based on 78 genomes.

MultiLoc2-26-10-2009 stand-alone version was used to predict 11 localizations of proteins in eukaryotic categories (fungi, invertebrate, plant, protozoa, vertebrate mammalian, and vertebrate other) with default option (MultiLoc2-HighRes). For input data of GOLoc subpredictor, results of InterProScan [6] version 5.7-48.0 were introduced to MultiLoc2 with ‘-go’ option. As MultiLoc2 program dependencies, Python version 2.7.3, LIBSVM [7] version 3.21, and BLAST [8] version 2.2.26 were used. This may result in slightly different prediction scores compared to those calculated by developer's online service. Eukaryotic organisms were divided into three groups: fungus, animal, and plant. In the prediction results, lysosome and chloroplast are not presented in fungus, and vacuole and chloroplast are not in animal, and lysosome is not in plant. There were program errors related to the absence of BLAST results in 2,646 sequences during the PhyloLoc run, and thus 7,717,340 protein sequences were processed from 7,719,986 input query sequences.

**Identification of orthologous groups**

Identification of orthology or paralogy is important for functional annotation of genome and facilitates studies on gene evolution and comparative genomics. To provide the information of orthologous groups for eukaryotes, we used the orthology detection program OrthoMCL [9]. OrthoMCL identifies eukaryotic ortholog group automatically from multiple species and makes clusters of orthologous groups across multiple taxa based on protein sequence similarity, using Markov Cluster algorithm [10].

OrthoMCL version 2.0.9 was used for grouping proteins into putative ortholog groups with default option. At family level, that is a level of biological classification of organisms, we chose 69 families which have at least 2 species, versions of genome assemblies, or database sources in the family, and then made putative ortholog groups for each family (Table S10). A total of 1,053,890 groups were made for 6,556,671 protein sequences of 312 species or assemblies in 69 families. We then identified the relationship between two proteins in the family. Because phylogenetic trees of families and evolutionary histories or distances of genes or proteins were not considered, we clarify that in-paralogs are not distinguished from out-paralogs in our results [11]. Also, because the same species which have at least 2 versions of genome assemblies or database sources in the family were used, statement of orthologs/paralogs are not correct. Thus, we classified the relationship as ‘homologs in other species’ and ‘homologs in the same species’.

**Table S13**.

| **Organismal**  **category** | **Family** | **No. of**  **species/assemblies** |
| --- | --- | --- |
| Fungi | Agaricaceae | 2 |
| Fungi | Ajellomycetaceae | 2 |
| Fungi | Clavicipitaceae | 2 |
| Fungi | Cordycipitaceae | 2 |
| Fungi | Coriolaceae | 2 |
| Fungi | Glomerellaceae | 2 |
| Fungi | Marasmiaceae | 2 |
| Fungi | Mycosphaerellaceae | 2 |
| Fungi | Nectriaceae | 2 |
| Fungi | Nosematidae | 2 |
| Fungi | Schizosaccharomycetaceae | 2 |
| Fungi | Sclerotiniaceae | 2 |
| Fungi | Sordariaceae | 2 |
| Fungi | Trichocomaceae | 2 |
| Fungi | Ustilaginaceae | 2 |
| Fungi | Unikaryonidae | 3 |
| Fungi | Chaetomiaceae | 4 |
| Fungi | Tremellaceae | 4 |
| Fungi | Arthrodermataceae | 5 |
| Fungi | Pleosporaceae | 5 |
| Fungi | Herpotrichiellaceae | 6 |
| Fungi | Aspergillaceae | 9 |
| Fungi | Debaryomycetaceae | 11 |
| Fungi | Saccharomycetaceae | 15 |
| Invertebrates | Onchocercidae | 2 |
| Invertebrates | Cionidae | 3 |
| Invertebrates | Culicidae | 3 |
| Invertebrates | Rhabditidae | 3 |
| Invertebrates | Apidae | 5 |
| Invertebrates | Drosophilidae | 13 |
| Plants | Cyanidiaceae | 2 |
| Plants | Volvocaceae | 2 |
| Plants | Bathycoccaceae | 3 |
| Plants | Euphorbiaceae | 3 |
| Plants | Malvaceae | 3 |
| Plants | Cucurbitaceae | 4 |
| Plants | Mamiellaceae | 4 |
| Plants | Rutaceae | 4 |
| Plants | Fabaceae | 7 |
| Plants | Rosaceae | 7 |
| Plants | Brassicaceae | 12 |
| Plants | Poaceae | 13 |
| Plants | Solanaceae | 15 |
| Protozoa | Saprolegniaceae | 2 |
| Protozoa | Sarcocystidae | 2 |
| Protozoa | Cryptosporidiidae | 3 |
| Protozoa | Theileriidae | 3 |
| Protozoa | Trypanosomatidae | 6 |
| Protozoa | Plasmodiidae | 9 |
| Vertebrates_mammalian | Pteropodidae | 2 |
| Vertebrates_mammalian | Tupaiidae | 2 |
| Vertebrates_mammalian | Camelidae | 3 |
| Vertebrates_mammalian | Cebidae | 3 |
| Vertebrates_mammalian | Delphinidae | 3 |
| Vertebrates_mammalian | Equidae | 3 |
| Vertebrates_mammalian | Felidae | 3 |
| Vertebrates_mammalian | Ursidae | 3 |
| Vertebrates_mammalian | Cricetidae | 5 |
| Vertebrates_mammalian | Vespertilionidae | 5 |
| Vertebrates_mammalian | Cercopithecidae | 6 |
| Vertebrates_mammalian | Muridae | 6 |
| Vertebrates_mammalian | Bovidae | 9 |
| Vertebrates_mammalian | Hominidae | 12 |
| Vertebrates_other | Alligatoridae | 2 |
| Vertebrates_other | Falconidae | 2 |
| Vertebrates_other | Tetraodontidae | 3 |
| Vertebrates_other | Phasianidae | 4 |
| Vertebrates_other | Poeciliidae | 5 |
| Vertebrates_other | Cichlidae | 6 |

**References**

1. Sonnhammer EL, Von Heijne G, Krogh A, editors. A hidden Markov model for predicting transmembrane helices in protein sequences. Ismb; 1998.

2. Emanuelsson O, Nielsen H, Brunak S, von Heijne G. Predicting subcellular localization of proteins based on their N-terminal amino acid sequence. Journal of molecular biology. 2000;300(4):1005-16.

3. Blum T, Briesemeister S, Kohlbacher O. MultiLoc2: integrating phylogeny and Gene Ontology terms improves subcellular protein localization prediction. BMC bioinformatics. 2009;10(1):1.

4. Nielsen H, Engelbrecht J, Brunak S, von Heijne G. Identification of prokaryotic and eukaryotic signal peptides and prediction of their cleavage sites. Protein engineering. 1997;10(1):1-6.

5. Emanuelsson O, Nielsen H, Von Heijne G. ChloroP, a neural network-based method for predicting chloroplast transit peptides and their cleavage sites. Protein Science. 1999;8(05):978-84.

6. Jones P, Binns D, Chang H-Y, Fraser M, Li W, McAnulla C, et al. InterProScan 5: genome-scale protein function classification. Bioinformatics. 2014;30(9):1236-40.

7. Chang C-C, Lin C-J. LIBSVM: a library for support vector machines. ACM Transactions on Intelligent Systems and Technology (TIST). 2011;2(3):27.

8. Altschul SF, Gish W, Miller W, Myers EW, Lipman DJ. Basic local alignment search tool. Journal of molecular biology. 1990;215(3):403-10.

9. Fischer S, Brunk BP, Chen F, Gao X, Harb OS, Iodice JB, et al. Using OrthoMCL to assign proteins to OrthoMCL‐DB groups or to cluster proteomes into new Ortholog groups. Current protocols in bioinformatics. 2011:6.12. 1-6.. 9.

10. Dongen SM. Graph clustering by flow simulation2000.

11. Sonnhammer EL, Koonin EV. Orthology, paralogy and proposed classification for paralog subtypes. TRENDS in Genetics. 2002;18(12):619-20.
